# Cryo-EM structure and B-factor refinement with ensemble representation

**DOI:** 10.1038/s41467-023-44593-1

**Published:** 2024-01-10

**Authors:** Joseph G. Beton, Thomas Mulvaney, Tristan Cragnolini, Maya Topf

**Affiliations:** 1 Leibniz Institute of Virology (LIV) and Universitätsklinikum Hamburg Eppendorf (UKE), Centre for Structural Systems Biology (CSSB), 22607 Hamburg, Germany; 2 Institute of Structural and Molecular Biology, Birkbeck, University of London, London, UK

**Keywords:** Computational biophysics, Software, Computational models, Electron microscopy, Statistical methods

## Abstract

Cryo-EM experiments produce images of macromolecular assemblies that are combined to produce three-dimensional density maps. Typically, atomic models of the constituent molecules are fitted into these maps, followed by a density-guided refinement. We introduce TEMPy-ReFF, a method for atomic structure refinement in cryo-EM density maps. Our method represents atomic positions as components of a Gaussian mixture model, utilising their variances as B-factors, which are used to derive an ensemble description. Extensively tested on a substantial dataset of 229 cryo-EM maps from EMDB ranging in resolution from 2.1-4.9 Å with corresponding PDB and CERES atomic models, our results demonstrate that TEMPy-ReFF ensembles provide a superior representation of cryo-EM maps. On a single-model basis, it performs similarly to the CERES re-refinement protocol, although there are cases where it provides a better fit to the map. Furthermore, our method enables the creation of composite maps free of boundary artefacts. TEMPy-ReFF is useful for better interpretation of flexible structures, such as those involving RNA, DNA or ligands.

## Abstract (web summary)

Cryo-EM is the go-to method for visualizing large, flexible biomolecules. Here, authors introduce a new Gaussian mixture modelling method for cryo-EM modelling tasks, including refinement, composite map generation and ensemble representation.

## Introduction

Cryo-electron microscopy (cryo-EM) can resolve the structure of biomolecules at an ever-improving resolution. Larger complexes can now be visualised as 3-dimensional density maps at near-atomic resolutions, and in various conformations. The interpretation of those maps often hinges on fitting atomic models of the different macromolecules present in the complex^1–3^. This procedure is often difficult and requires the user to provide accurate models, and a well-estimated resolution (which can vary at different parts of the map). Pre-existing experimental or predicted atomic models may be in a different conformation, and converging to a well-fitted one may require significant sampling.

Several methods are commonly used for this procedure. To improve the map fit, the map can be treated as a scalar field, for which a gradient can be used as a force^4,5^. Optimisation of the position against the correlation coefficient (CCC) has also been proposed^6^, or by Bayesian expectation-maximisation (EM) against the density observed in the map^7,8^. The sampling itself is usually based on either molecular dynamics (MD)^4,9^, minimisation^10^, normal mode analysis and/or gradient following techniques^11,12^, or Fourier-space-based methods^2^. Manual inspection and modification of the structure, or targeted sampling for specific parts of the structure, are also common, especially at high resolutions^13–15^.

Molecular dynamics-based refinement methods have the advantage of wider sampling but may result in locally distorted structures. This can usually be fixed by either clustering the resulting data^9^ or by minimising the structures at the end of the run^6^. The use of a force field (such as CHARMM^16^ or AMBER^17^) have the added benefit of ensuring that clashes are generally absent from the structure since they include parameterised van der Waals repulsion terms.

Virtually all methods rely on blurring the model (globally or locally)^18^ to compare against the experimental map, which poses an additional challenge for maps of flexible systems that will often exhibit significant resolution heterogeneity between flexible and rigid regions. This heterogeneity in the map can also result from adding up density maps from different reconstructions (e.g., result of multibody or focused refinement) into a so-called composite map^19,20^. However, a systematic way to combine multiple maps into a composite map has not been proposed yet.

Flexibility is intrinsic to biomolecular systems, which presents a challenge for methods that tend to rely on a single structure representation. Methods using a population of models^21–26^ can provide an improved understanding of the fit between map and models^27^. Mixture modelling is a powerful framework to represent arbitrary density probabilities comprising several parts: by iteratively estimating the model parameters, and then re-computing the expected distribution, a (locally) optimal model can be generated^8^. We use this approach to estimate both the local spread of density around atomic positions and the background noise level.

**Fig. 1:**
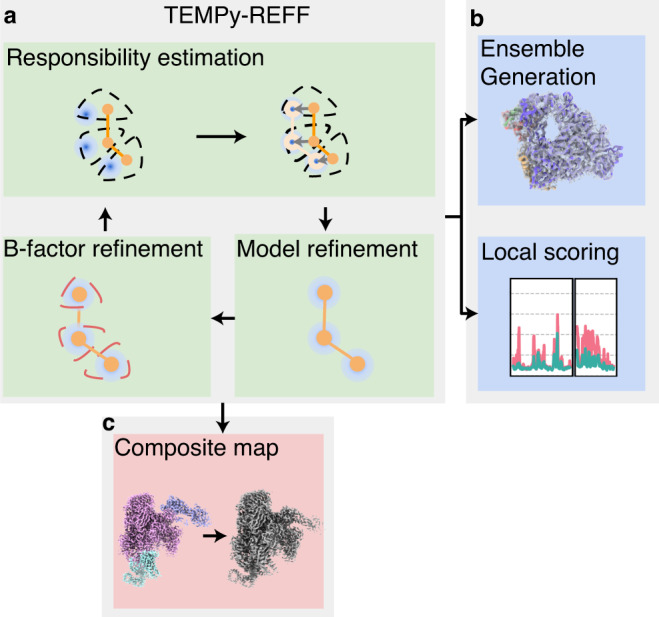
Flow chart summarising the steps in the TEMPy-ReFF algorithm. **a** The EM (Expectation-Maximisation) algorithm. Responsibility is an estimation of the part of the data that is represented by a given component in the mixture. New parameters (the mean and variance of each component corresponding to the position and B-factor value) for each component (e.g., for each atom) are then re-estimated using this responsibility and the experimental data. **b** After refinement, an ensemble can be generated based on the local variance; local scoring provides a view of the quality of fit of all regions of the map, irrespective of the local resolution. **c** By considering the sum responsibility of all the atoms in a chain, we obtain a natural expression of the part of a map represented by a given chain. This can be used for composition.

Here, we propose TEMPy-ReFF (REsponsibility-based Flexible-Fitting)—an MD-based refinement guided by an EM scheme that uses a Gaussian Mixture Model (GMM) to provide self-consistent estimates for the atomic positions and local B-factors (Fig. 1). We show that the method can accurately treat maps with highly heterogeneous resolution. To assess the quality of the refined models, we have developed a measure that estimates the quality-of-fit of every residue to the local density and allows us to compare the fit of different parts of the model in regions of varying resolution. We demonstrate on a large dataset (from the CERES database http://cci.lbl.gov/ceres and additional cases from the Protein Data Bank^28^ (PDB) and Electron Microscopy Data Bank^29^ (EMDB)), that our approach produces single fits of similar quality compared to state-of-the-art methods, such as Phenix^30^ although it can sometimes provide improved ones. Importantly, we show that our B-factor refinement approach not only allows for the generation of an ensemble of atomic models to better represent the density information but also enables the generation of more reliable composite maps.

## Results

### Mixture modelling applied to refinement

We have developed a method based on a GMM (using one Gaussian per atom and a uniform background term) to represent the estimated contribution of various parts of a model to the experimentally observed intensity. The Gaussians are fitted to the model in a self-consistent way, such that their summed contributions represent a (locally) optimal fit to the density. The intensity attributed to a Gaussian, or a sum of Gaussians, can be used to estimate their importance in representing a specific part of the map density. For example, by summing the Gaussians for atoms from a given protein chain, it is possible to determine which part of the map is best represented by this chain, or other chains, or are part of the general background noise in this map. Those weighted contributions (termed responsibilities in GMM literature) allow us to perform a variety of tasks that are commonly performed on cryo-EM maps (described in Fig. 1): fitting an atomic model to the map, segmenting the map into several parts, each representing a distinct entity (for example, a distinct subunit in a protein complex), or combining focused maps into a single overall composite map, with optimal weights of the focused maps.

Although GMM approaches have been successfully employed before, this was usually a coarse-grained representation of the overall model and map^7,8,31^. By describing each atom as a Gaussian point spread function, a link between map and model is directly established: the intensity of each voxel is a direct sum of the contribution of each atom, as a function of its position and B-factor. It is important to note that we define each atom’s “B-factor” as the sigma of its respective Gaussian in the GMM. Additionally, the formalism used here does not require the use of Gaussian distributions, and alternative descriptions for the individual atomic contributions could be considered.

The responsibility calculation has several benefits: for regions of the map that are close to multiple parts of the structures, the mixture model allows for uncertainty in the assignment of the density. This soft-mixing improves the convergence of the refinement, by making it easier for structural elements to slide towards regions of density that are a better fit, even if they are currently fit to a high-density region of the map. The calculation is also self-consistent, as is empirically demonstrated below: changes in the initial position and B-factor assignment for the structure result in identical or similar fit for a wide range of initial values.

### Ensemble generation based on B-factors

**Fig. 2:**
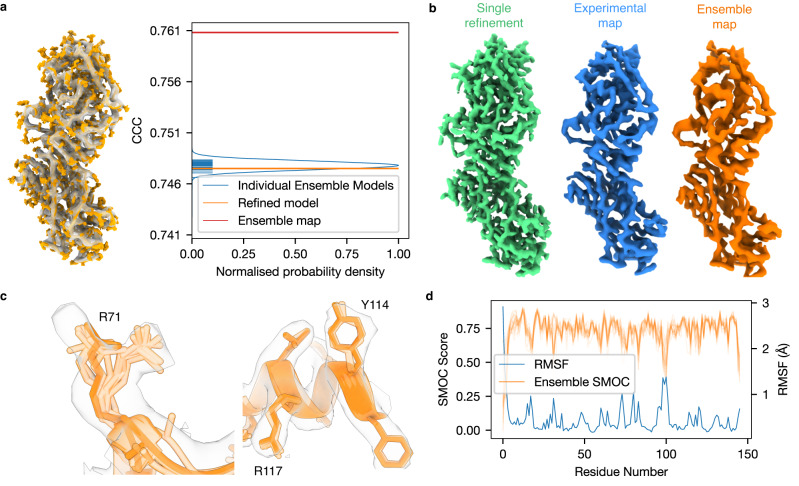
Ensemble representation of cryo-EM models. **a** Depiction of structure ensemble (orange), along with the map (transparent grey); a plot of the CCC of each individual model in the ensemble is shown (blue horizontal lines from *y* axis), as well as the ensemble map (red). **b** depiction of a single-model map (green), experimental map, and our computed ensemble map at contour level 0.02. **c** Differences in the ensemble for different residues, for the ensemble of the Methionine Transporter (PDB ID: 7MC0, EMDB ID: 23752): for residue R71 (left) the ensemble is more widespread, and the side-chain density is more spread out into two peaks, each populated by parts of the ensemble. For high-resolution portions of the map shown on the right side, for example, R117 and Y114, the ensemble is highly constrained, and the side-chain density is well-defined. **d** SMOCf plot (shown in orange) and RMSFe (shown in blue) for each structure in the ensemble for the Faba bean necrotic stunt virus (PDB ID: 6S44, EMDB ID: 10097, map resolution 3.3 Å); the RMSF and SMOCf score are clearly anticorrelated.

Our GMM representation models the local ambiguity within cryo-EM maps by tuning the B-factor of each atom. We reasoned that we could leverage this information to generate an ensemble of models that more accurately represents the variety of conformations that are compatible with the map. Models were randomly generated by perturbing the positions of atoms, based on their B-factors, followed by local L-BFGS^32^ minimisation (with OpenMM^33^) to locate close-by structures that were compatible with the data^34^. Ensemble maps were computed by averaging the simulated maps obtained for all sampled structures in the ensemble (Fig. 2).

We first assessed the accuracy of B-factor assignment in TEMPy-ReFF. While the B-factor optimisation is intended to be used together with position refinement, it is useful to test it independently by optimising the B-factors while keeping the atomic positions fixed. We found that for all cases we tested, the map-model CCC improved significantly when taking into account the refined B-factors for map simulation (Supplementary Table 1). The average B-factor convergence is shown in Supplementary Fig. 1., along with the corresponding change in the CCC using the examples of Faba bean necrotic stunt virus (FBNSV) (EMD-10097, 3.2 Å resolution, PDB ID: 6S44) and the SARS-Cov-2 RNA-dependent RNA polymerase (EMD-30127, 2.9 Å resolution, PDB ID: 6M71). The distribution of the B-factors is similar to that of B-factors obtained from the deposited models (Supplementary Fig. 1b). Furthermore, the B-factor assignment is robust: we found that two refinements starting at initial values that differed by a factor of 5 converged to a similar solution (Supplementary Fig 2). Finally, when we updated the atomic positions, we observed changes in the B-factors, this is a feature of the change in coordinates (as the two are not independent) (Supplementary Fig. 3).

Next, we investigated the use of our calculated B-factors in ensemble generation (Fig. 2). The best-fitted model for rotavirus VP6 (EMD-6272) at 2.6 Å appears as only one solution among many in the generated ensemble (Fig. 2a). On the other hand, the ensemble average map exhibits a much higher quality-of-fit to the experimental map than any single model (Fig. 2a, b, Supplementary Fig. 4). Intriguingly, the ensemble map resembles more closely the experimental map (Fig. 2a). We determine the optimal number of models in an ensemble by calculating the CCC with the ensemble map generated from an increasing number of models (Supplementary Fig. 5). A visual comparison between the single TEMPy-ReFF refined model and the ensemble is shown in Fig. 2c where insets of residue fit show the source of improvements: the density for an arginine (R71 from chain A) could be explained by positioning the side chain in two alternate conformations. The structures in the ensemble populate both possible conformations (Fig. 2c, left inset). In contrast, the ensemble of models is much more tightly clustered in well-resolved portions of the map, for example, residues R117 and Y114 from chain A (Fig. 2c, right inset). We also found, using the capsid protein from the Faba bean necrotic stunt virus (PDB ID: 6S44, EMDB ID: 10097, map resolution 3.3 Å), that the per-residue SMOCf^35^ score (averaged between all ensemble members) showed a strong anti-correlation with the RMSF between the ensemble measures (Pearson’s coefficient −0.81, Fig. 2d).

### Benchmarking structure refinement

We assessed the quality of TEMPy-ReFF model refinement using a large dataset of 229 models taken from the PDB (see Methods) with corresponding maps at resolutions between 1.8 and 5 Å. We compared the CCC, MolProbity^36^, and CaBLAM^37^ scores before and after refinement. We benchmarked our method against the deposited PDB models as well as CERES^38^ (see Methods), which is an automated Phenix^30^ model re-refinement programme for cryo-EM maps at resolution ≤5 Å.

**Fig. 3:**
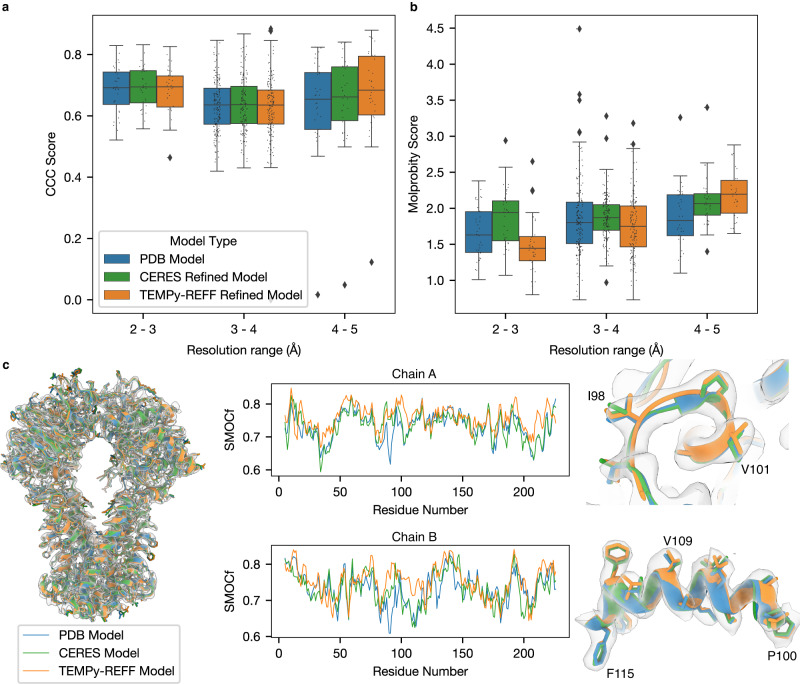
Refinement of the CERES benchmark. **a** Benchmark comparison using CCC, between the initial (PDB-deposited) models (blue), the CERES re-refined models (green), and TEMPy-ReFF refinement-based model (orange), separated by resolution bands of 1 Å. We evaluated *n* = 229 individual models. The central line in each boxplot defines the median value, the bounds of each box define the upper and lower quartiles and the whiskers define 1.5 times the interquartile range (IQR). Outliers (points outside this 1.5*IQR range) are marked with rhombus symbols. The individual score for each model is marked with a black point. **b** Benchmark comparison of the same 229 models using MolProbity score, the colouring and layout of the boxplot is the same as in **a**. **c** Comparison between the refinement of the ABC methionine transporter (PDB ID: 7MC0, EMDB ID: 23572, resolution 3.3 Å) with TEMPy-ReFF and the corresponding models from PDB and CERES. For all subpanels the colouring matches that used in **a**. The left panel shows the overlaid models within the cryo-EM map, which is rendered as a transparent surface. The central panels show the SMOCf scores for residues from chains A (upper panel) and B (lower panel). The left-hand panels show zoomed-in views of sections of chain A (upper panel) and B (lower panel) as highlighted in the respective SMOCf plots with black outlined boxes.

We observed, overall, similar performance between TEMPy-ReFF and CERES based on map-model similarity (CCC) and geometric model quality scores (MolProbity, CaBLAM, clash score) (Fig. 3, Supplementary Table 2). The average CCC scores for refined models from maps with a resolution range of 3–4 Å from TEMPy-ReFF (median: 0.633, mean ± std: 0.627±0.101) and CERES (median: 0.636, mean ± std: 0.637±0.087) were very similar (Fig. 3a). We only observed improved average CCC scores from TEMPy-ReFF refinements for models refined in maps at 4–5 Å resolution (mean CCC ± std from TEMPy-ReFF: 0.672±0.148, CERES: 0.651±0.147). However, we observed improved (lower) average MolProbity scores in many TEMPy-ReFF refined models. Specifically, the MolProbity scores for TEMPy-ReFF refined models from the highest resolution maps (<3 Å), outperformed both CERES and models obtained from the PDB. Additionally, we noted a smaller improvement in MolProbity scores for models in the 3–4 Å resolution range. This was largely due to the almost total absence of clashes in TEMPy-ReFF refined models (Supplementary Table 2). However, we noted more CaBLAM outliers in TEMPy-ReFF refined models. Further, we observed a higher correlation between MolProbity score and map resolution (i.e., increasing MolProbity score as map resolution worsens) for TEMPy-ReFF refined models compared to those obtained from the PDB and CERES (Supplementary Fig. 6). This might be due to geometric restraints that are commonly applied in other refinement software, including in CERES^38^, but not in TEMPy-ReFF, where the geometry of the model is derived from the energy function and the MD force field.

We examined the local fit quality using SMOCf for one example from our benchmark: the ABS methionine transporter, solved at 3.3 Å (PDB ID: 7MC0, EMDB ID: 23572). This example showed that local model fit for the TEMPy-ReFF refined model was similar overall, relative to those from the PDB and CERES (Fig. 3c). Some parts of the TEMPy-ReFF models showed better fit, and others poorer. This was perhaps unsurprising, given the overall similar performance across our benchmark at this resolution range (Fig. 2a). In areas where we did observe better local fit for TEMPy-ReFF refined models, this was apparently due to subtle changes in the positioning of the backbone and the orientation of side chains (Fig. 3c).

We next investigated the degree of structural rearrangement that was possible during TEMPy-ReFF refinement. We identified structures deposited in the EMDB/PDB of which two separate conformations were identified. First, we analysed two structures of the Atm1 ABC transporter, in an open and closed conformation (EMDB IDs: 13613, 13614 at 3.3 and 3.2 Å resolution, respectively and corresponding PDB IDs: 7PSL, 7PSM, respectively)^39^. We observed that large structural rearrangements (e.g., rotation of whole domains) would be required to refine the structure of closed conformation into the cryo-EM map of the open conformation (i.e., to refine the 7PSM into EMD-13613). Despite an increase in CCC from 0.15 to 0.31, refinement with TEMPy-ReFF was not able to reproduce the structure of the open conformation, presumably because the model became stuck in local minima (Supplementary Fig. 7a). In a previous study, we developed a method that combined density-guided-refinement (which is similar to MDFF^40^), with the hierarchical application of rigid-body restraints calculated using RIBFIND2 (version 2.0). This method was able to correct large structural changes in RNA complexes^41–43^. We applied this method to the refinements of Atm1 and were able to successfully refine the model from the closed conformation into the open cryo-EM map (Supplementary Fig. 7a). The CCC was 0.34 after rigid-body refinement. We noticed some errors remained in the model, such as slightly incorrect placing of ɑ-helices and amino acid side chains. To fix these issues, we ran an extra round of refinement using TEMPy-ReFF, which further improved the model to a final CCC of 0.54. We observed a similar outcome for refinement of the open conformation CGT ABC transporter^44^ (EMDB ID: 14843, PDB ID: 7zo8) into the cryo-EM map for the closed conformation (EMDB ID: 14844, PDB ID 7zo9): refinement was only successful when combined with the application of hierarchical RIBFIND2 restraints (Supplementary Fig. 7b). Thus, we conclude that TEMPy-ReFF refinement, without additional rigid-body restraints, is best suited for refinement that requires local changes in the model, for example, arrangement of secondary structure elements and positioning of side-chains.

### B-factor weighted composite maps

We hypothesised that our GMM approach for model representation could be applied to generating composite maps, where one combines multiple, potentially overlapping, reconstructions of the same complex into a single map. This can be viewed as an inverse of the mixture modelling problem, where the intensity contributions of each component map must be correctly mixed together to produce an accurate composite map. We achieved this using our GMM representation to calculate responsibilities for every voxel in each component map (Eq. 9), such that portions of component maps that corresponded to atoms with lower B-factors were assigned the highest responsibilities. These responsibilities acted as weights for combining the component maps (Eq. 10). Our approach has several advantages: because the responsibility decays smoothly, there are no seams within composite maps and areas where the assignment would be uncertain are treated as such, and the density will not be arbitrarily assigned to a specific model or submap.

**Fig. 4:**
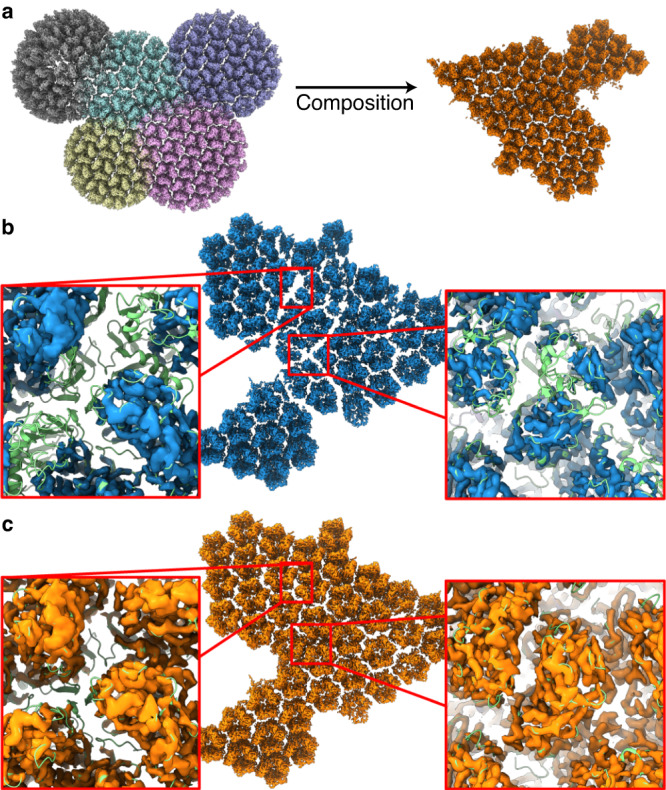
Using TEMPy-ReFF for map composition. **a** Composition of 5 component maps (EMD-34227, 34229, 34230, 34235, 34236) shown in their overlapping position on the left, combined to create the composite map shown on the right. **b** Composite map of the Singapore grouper iridovirus capsid (EMD-34815), shown as a blue surface rendering. In order to simplify visual comparison, we masked the original map such that only density around the fitted model (PDB ID: 8HIF) is shown. The deposited composite map retains some artefacts at the borders between the, approximately circular, component maps, where the map density is less intense. This is highlighted in the insets, which also show the fitted model, coloured green. **c** Composite map, shown as an orange surface rendering, produced using the responsibilities computed by TEMPy-ReFF as weights for each component map. The insets show the map density with the model, again shown in green, at the same location as shown per **b**. Clearly, the artefacts are no longer present.

We evaluated our approach on a composite map of the Singapore grouper iridovirus capsid (EMD-34815) (Fig. 4). This map is composed of 5 component maps, with each overlapping significantly with at least 2 other component maps (Fig. 4a), using Chimera^45,46^. Circular artefacts were visible in the deposited map, which occurred at the edges of the individual component maps, including at areas of the map containing a fitted model (Fig. 4b, Supplementary Fig. 8a). After generating a composite map using our responsibility-weighted approach, we found no visually distinguishable artefacts at equivalent locations in our composite map (Fig. 4c). This was reflected in a general increase in correlation between Fourier components of the TEMPy-ReFF composite map and the deposited model, compared to between the deposited map and model (Supplementary Fig. 8a). Additionally, the CCC between the model and TEMPy-ReFF composite map improved to 0.79, compared to 0.71 for the deposited map.

We extended our evaluation to composite maps which did not include visually obvious reconstruction artefacts by reproducing the composite map of RNA polymerase II (EMD-12969), composed from 3 separate maps (EMD-12966, EMD-12967, EMD-12968)^47^. Here, we again see a general increase in correlation between Fourier components in the TEMPy-ReFF composite map and the model, as well as an increase in the model CCC score to 0.61, from 0.51 for the deposited map (Supplementary Fig. 8b).

### Case study 1: yeast RNA polymerase III elongation complex

We explored the effectiveness of the TEMPy-ReFF approach in more detail by refining the model of yeast RNA polymerase III elongation complex (PDB ID: 5FJ8). The corresponding cryo-EM map (EMD-3178) was resolved at a global resolution of 3.9 Å^48^. A brief observation of the deposited model suggests that it is well-fitted to the cryo-EM map: we computed the CCC, using ChimeraX, as 0.58. The validation statistics presented in the PDB are reasonable; clash score of 14, Ramachandran outliers 1.1% and side-chain outliers 2.1%, with an overall MolProbity score of 2.8.

**Fig. 5:**
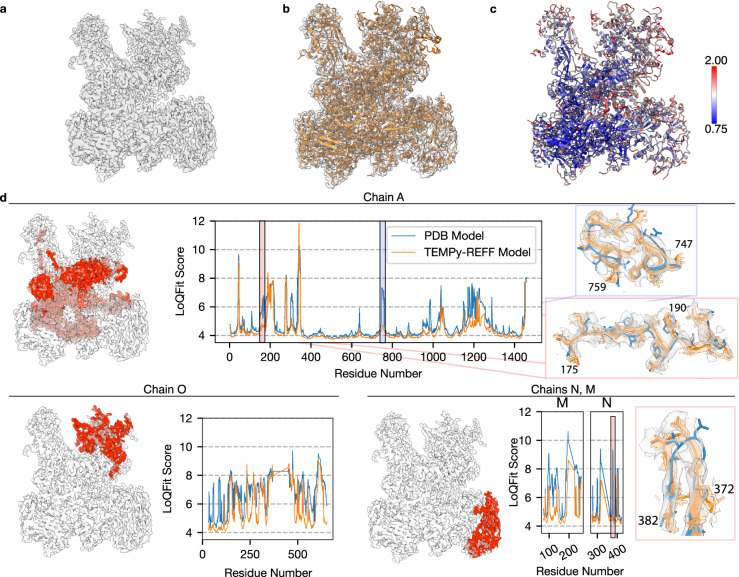
Case study of RNA polymerase III elongation complex. **a** The deposited 3.9 Å cryo-EM map of the RNA polymerase III elongation complex (EMD-3178). **b** The TEMPy-ReFF refined model of the RNA polymerase III complex deposited structure (PDB ID: 5FJ8) shown within the cryo-EM density. **c** the TEMPy-ReFF refined model (right) coloured according to the refined B-factors. **d** LoQFit scoring of individual chains from the RNA polymerase III complex, with the scores for the starting model (obtained from the PDB) shown in blue, and for the TEMPy-ReFF refined mode shown in orange. The position of these chains within the original cryo-EM map are highlighted in red. Insets show several regions before and after refinement coloured as per the LoQFit plots, with the ensemble of models shown in transparent orange.

The TEMPy-ReFF refined model had an improved correlation with the map, with a single-model final CCC of 0.62, whilst the ensemble map had a CCC of 0.70. The MolProbity score remained essentially unchanged at 2.7. A representation of the model, as well as the quality-of-fit for multiple chains, is shown in Fig. 5.

We next applied the TEMPy LoQFit score (see Methods) to locally assess the improvement of our TEMPy-ReFF refined model, versus the deposited model. Here, we only use the single-refined model from TEMPy-ReFF to ensure fair comparisons. We visualise the LoQFit score at each residue in both models using 2D plots (Fig. 5). The average LoQFit score for the deposited model was 5.1 Å, and model agreement was particularly high in chains A and B at the central regions of the model and map, where the average LoQFit score was 4.6 and 4.5 Å, respectively. However, even in these regions we observe peaks in the LoQFit score, consistent with poorer model fit, such as those seen around residues 192–210 and 745–759 in chain A (Fig. 5d), as reflected in the higher B-factors in this region (Fig. 5c). In addition to this, we identify extended regions of poorer model fit, generally occurring within chains that lay at the edge of the complex in solvent-exposed regions with poorer resolution, including chains M and N (Fig. 5d). In these chains the average LoQFit score was 6.6 and 5.4 Å, respectively, reflecting the lower map resolution (and correlating with high B-factors), as well as poorer model fit in the deposited model. Refinement with TEMPy-ReFF resolved many of these poorer fitting regions: the average LoQFit score for the refined model improved to 4.6 Å, and we observed significantly better model fit at lower resolution regions of the map. The average LoQFit score for chain O improved to 5.7 Å in the refined model (from 6.8Å, Fig. 5d), and in chains M and N the average LoQFit score improved to 5.1 and 4.5 Å after refinement. We investigated the significance of these changes in the LoQFit score. Firstly, we observed a close correlation between the LoQFit score and the local resolution at the equivalent position within a cryo-EM map (Supplementary Fig. 9). Secondly, we benchmarked LoQFit against other common local scoring functions, Q-score and SMOC, as well as our B-factor refinement. For Q-score and B-factors, we used the residue average (Q-score_avg_), for comparison. To do this benchmarking, we measured the LoQFit, Q-score_avg_, SMOCf and B-factors for 50 models refined by TEMPy-ReFF, and investigated the correlation between LoQFit and the other scoring functions via Pearson’s correlation. This revealed a significant, inverse, correlation between LoQFit and Q-score_avg_ (−0.62 Pearson’s correlation across all examples), and a significant correlation between LoQFit and the residue average B-factor (0.64 Pearson’s correlation across all examples). We observed a much less significant correlation with the SMOCf score (0.32), which varied much more significantly across the examples we tested, compared to the correlation between LoQFit and Q-score_avg_ and average B-factor (Supplementary Fig. 10). This was unsurprising, given the previously reported lack of correlation between the Q-score and SMOCf^49^.

### Case study II: nucleosome-CHD4 complex structure

**Fig. 6:**
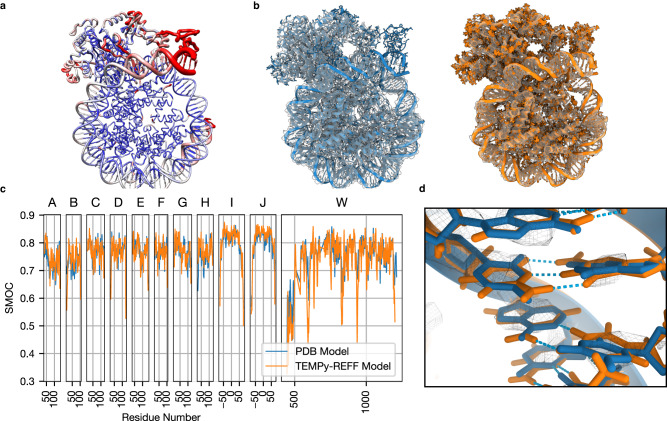
Case studies of Nucleosome-CHD4 complex. **a** A nucleosome structure in complex with chromatin remodelling enzyme CHD4 (EMD-10058, PDB ID: 6RYR) is shown (worm representation), with the width proportional to the TEMPy-ReFF refined B-factor, and colour based on local resolution (computed with ResMap). **b** Deposited model (left, blue) and the ensemble of models and ensemble map calculated with TEMPy-ReFF (right, orange), shown inside the cryo-EM map (transparent grey). **c** SMOCf plot for each chain. The deposited model is shown in blue, and the TEMPy-ReFF model is shown in orange. **d** Zoom-in on some of the DNA base pairs (chain I/J, base pair 54) fitted in the map (mesh representation). The deposited model is shown in blue, TEMPy-ReFF model in orange and hydrogen bonds are indicated in cyan.

The nucleosome is a large nucleoprotein present in the nucleus, which is the primary effector in the compaction of DNA. High-quality reconstructions have been obtained, but its dynamic nature and strained DNA strands wound around the histone proteins make it a challenging system to obtain a good structural model. We apply TEMPy-ReFF to refine the model associated with map EMD-10058^50^ (PDB ID: 6RYR) (Fig. 6a–d). The deposited cryo-EM map clearly suffers from very variable resolution (range: 3–10 Å, see Supplementary Fig. 9), which affected the quality-of-fit of the deposited model (Fig. 6a). Following refinement, the local details of the map are well respected, especially showing improvement in the DNA structure, as reflected by the SMOCf score (chain I and J, Fig. 6c). Nucleic acids are often present in biomolecular complexes resolved by Cryo-EM, and refining their geometries with respect to the map is an important part of model refinement. In the deposited model, local deformations pull the bases slightly away from the density, and from the expected geometries to allow hydrogen bond formation. Our automated refinement pulls them back, forming hydrogen bonds in the process (Fig. 6d). After refinement, the LoQFit and the local resolution follow similar trends (Supplementary Fig. 9), indicating the model is well fit to the map. This case study also further demonstrates how the ensemble map calculated with TEMPy-ReFF has greater similarity with the experimental map than a single model (either the deposited model or a single-refined model).

### Case study III: SARS-CoV2 RNA polymerase and AlphaFold2

To refine a model into an experimental cryo-EM map, an initial model is needed. Although building a reliable model directly from the map is sometimes possible, in most cases, this cannot be done reliably as the resolution is not sufficient to allow a reliable assignment of every atomic position. In such cases, a starting model can be obtained using deep-learning-based ab initio tools, such as AlphaFold2^51^ or RosettaFold^52^. These programmes are frequently able to create very high-quality protein models^53^. The predicted lDDT score^51^ (plDDT) is also an excellent tool to decide which part of the model can be reliably kept, and which may not be correctly predicted, due to flexibility or lack of known homologous sequences and structures.

**Fig. 7:**
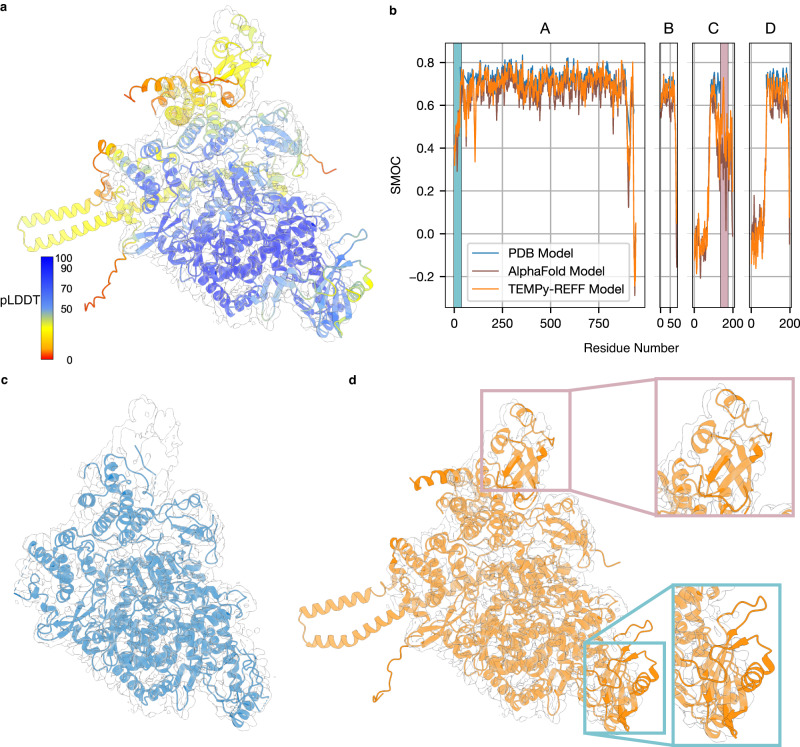
Case studies of SARS-CoV-2 RNA polymerase (AlphaFold2 model refinement). **a** AlphaFold2 predicted structure, with the colouring indicating the plDDT confidence measure (blue means higher confidence, red means lower confidence), fitted in the deposited map (EMD-30127, grey) **b** SMOCf plot of the AlphaFold2 (shown in blue) and TEMPy-ReFF refined model (shown in orange). The regions highlighted in grey and pink (correspond to inset regions in Fig. 7d) contain residues that are not present in the deposited model but are present in the AlphaFold2 model and are well-fitted to the map. **c** Deposited model for the SARS-CoV-2 RNA polymerase (PDB ID: 6M71, blue) fitted in the deposited map (transparent grey). Unassigned regions are visible, at the top and bottom right of the map. **d** TEMPy-ReFF model (orange) obtained by refining the AlphaFold2 prediction in the deposited map (transparent grey). Newly modelled regions that fit in the density (as in Fig. 6h) are shown with coloured squares.

To assess the capability of our method to refine such a model, we used AlphaFold2-Multimer^54^ to create a model of the SARS-Cov-2 polymerase. We used the polymerase sequence (UNIPROT ID: P0DTD1, residues 4393–5324), with non-structural proteins 7 (UNIPROT ID: P0DTD1, residues 3860–3942) and 8 (UNIPROT ID: P0DTD1, residues 3943–4140). We only used templates present in the PDB at least a year earlier than the deposition date of the deposited model (PDB ID: 6M71)^55^. The predicted model was refined into the SARS-Cov-2 polymerase cryo-EM map at 2.9 Å resolution (EMD-30127) (Fig. 7). The resulting model (Fig. 7d) is highly similar to the deposited model (Fig. 7c) at most residue positions, which was modelled using Chimera^46^, Coot^14^, and Phenix^30^. However, more intriguingly, using a SMOCf plot, we show that some residues that were not present in the deposited structure^55^ can actually be placed into the map, with fitting scores much greater than chance (Fig. 7c, d).

## Discussion

We have presented TEMPy-ReFF, an MD-based atomic structure refinement method, which is driven by the local features of a cryo-EM map using a mixture model with an error term, to account for the noise in the map. Our approach naturally incorporates both position and B-factor estimations in the same framework. This information is essential to represent the local variability around atomic positions. We conducted comprehensive testing on a substantial dataset comprising 229 cryo-EM maps sourced from EMDB, spanning resolutions from 2.1–4.9 Å and their respective PDB and CERES atomic models. On a single-model level, TEMPy-ReFF achieves performance similar to the CERES re-refinement protocol, and in some instances, outperforms it by providing a more accurate fit to the map.

Currently one of the greatest challenges in model building into cryo-EM maps is evaluating the quality-of-fit in a system not described by a single resolution value, but rather varying local resolution. We address this challenge using B-factor estimation. We find, as previously shown^21–23,25,26^, that an ensemble of equally well-fitted models represents this local variability better than a single model. However, we go one step further, by showing that an ensemble map calculated from these models, provides a better representation of the experimental map, in comparison to a traditional simulated map (which is typically generated from a single Gaussian function per atom) (Fig. 2a). This is showcased in Fig. 2c, where a potential double occupancy site for an arginine necessarily requires more than one model to be correctly represented. The improvement is also evident in regions of lower local resolution (Supplementary Fig. 4), which may indicate an inherent local flexibility of the structure, although this cannot be easily deconvolved from the blurring due to optical factors^56^, or image processing approaches.

Ensemble methods have been common practice in the NMR community and have been suggested as a way of dealing with the uncertainty in the data^22,23,34^. This has also been demonstrated previously for X-ray crystallographic data^57^, and we similarly observe a plateau as more models are added to the ensemble (Supplementary Fig. 5). Furthermore, when analysing the differences on a local level (for example at the residue level) using a distance measure (such as the RMSF), we observe that the local-fit-quality (using SMOCf) correlates well with those differences (Fig. 2d).

Overall, our automated refinement procedure is computationally efficient: computation time scales approximately linearly with map and model size (Supplementary Fig. 11). The resultant models are well-fitted to the cryo-EM map, based on the CCC. Without the ensemble representation of the fitted models, the local and global model-map fit score is comparable with those from Phenix (as represented by our comparison with CERES results). We also observed that TEMPy-ReFF refined models have typically as good, or better, MolProbity scores, compared to those from CERES and the PDB, across our benchmark (Fig. 4b). However, the correlation between resolution and MolProbity score was stronger for TEMPy-ReFF refined models, compared to those from CERES (Supplementary Fig. 6). This difference is likely due to a different application of explicit structural restraints in CERES, compared to TEMPy-ReFF. Our refinement procedure does not include any specific restraints, for example, to reduce Ramachandran or rotamer outliers. Rather, models refined by TEMPy-ReFF are implicitly restrained by the balances of forces applied to the atoms by the force field. This should produce models with appropriate geometry, assuming the fitting force from the GMM is appropriately balanced within the MD force field. Indeed, the generally good MolProbity scores obtained in our benchmark (Fig. 4b) show this to be an appropriate approach. In particular, we noted that TEMPy-ReFF refined models virtually never contained significant clashes (Supplementary Table 2). However, many refinement programmes, including those used for CERES models, do apply geometric restraints (e.g., to eliminate phi/psi outliers). Based on our results, it seems that, broadly, these restraints favour reduced CaBLAM outliers, which are typically better for PDB/CERES models, at the expense of clash scores, which were consistently worse in PDB/CERES models compared to those from TEMPy-ReFF (Supplementary Table 2). We also show that TEMPy-ReFF refinements of nucleic acids can simultaneously improve the fit to the cryo-EM data and the chain geometry (Fig. 6a–d).

Since 2018, deposition of composite maps has been increasing significantly due to a growing number of macromolecular assemblies for which focused maps for different assembly subunits are obtained (often due to conformational flexibility). Some methods have been proposed to compose such maps^20^, however, there is currently no systematic way to evaluate this. Here, we provide a self-consistent way to perform this procedure. Our approach has the advantage that the responsibility decays smoothly, i.e., there are no seams between segmented maps, or within composite maps: areas where the assignment would be uncertain are treated as such. However, the method also has some drawbacks, the clearest of which is that errors in modelling will result in errors in composition, and that the maps must be aligned manually, or using another software, prior to composite map generation with TEMPy-ReFF.

Finally, we show that our refinement protocol can take advantage of recent developments in the field of structure prediction^51,52^. Starting refinements from AlphaFold2^51,52^ models is not only possible, it gives results on par with manual refinement (despite using an automated procedure) and highlights that better and more complete models can be obtained by using our automated refinement approach, including more residues that are sustained by the map information (Fig. 6e–h). However, we note that models that contained large errors required the application of rigid-body restraints for effective refinement (Supplementary Fig. 7). For these refinements, the TEMPy-ReFF GMM-based (unrestrained) refinement still played an important role in correcting minor errors that existed after rough refinement with rigid bodies. It is difficult to define an exact transition point at which rigid-body refinement, instead of unrestrained, is required for a given model, and this currently requires user intervention. However, we envisage a flexible and automated combination of these approaches could pave the way for more reliable, and reproducible model building, where alterations in refinement protocols can be objectively and continuously assessed^53,58^.

Further work will be needed to understand the impact of ensemble model representation, and how to use such an approach in assessing model-map fit quality, especially for inherently flexible protein assemblies observed by cryo-EM. In this work, we explore how ensembles can be derived from local resolution information using our GMM interpretation of the experimental data. Although we are able to derive ensembles that improve the overall correlation with cryo-EM map, the model is admittedly simplistic. Assumptions that the Gaussians are isotropic and that resolution fluctuations are a result of conformational heterogeneity are approximations. Indeed, future work needs to be able to disentangle resolution heterogeneity due to reconstruction and imaging artefacts from that caused by atomic displacements and structural variation. It is foreseeable that this will require an end-to-end approach where more information from reconstruction and the underlying 2D micrographs are used to address these challenges. Despite these limitations, we see this work as an important step, particularly in the field of drug discovery, where, the docking of candidate compounds is dependent on the local environment, and local errors or variability can significantly alter the results. Providing multiple models of cryo-EM maps from near-atomic to medium-resolution will allow more reliable predictions of ligand poses, thereby opening a window to many potential drug targets in medium-resolution cryo-EM maps.

## Methods

### Refinement algorithm

Given an atomic model, which can be described as a set of atoms each possessing a coordinate *x*, a B-factor *B* and an atomic numbers *Z*, the aim is to optimise these positions and B-factors to best model the experimental data. The refinement algorithm is inspired by the EM approach for GMMs^59^. Here, atoms are represented as Gaussians with the centre of mass and B-factor represented by the mean of the Gaussian and sigma, respectively. Per the standard EM algorithm, we first compute the expected (simulated) map given the estimated atomic properties. A maximisation step is then performed to optimise the atomic properties. Traditionally, the maximised properties would be fed back to the expectation step and the EM process would be repeated until convergence. In order to incorporate stereochemical and physical information, we deviate from the standard EM algorithm: Rather than feed the maximised atomic properties back into the next expectation step we compute a force that biases atoms towards the optimised coordinates in an MD simulation. The algorithm is summarised below:

- Perform maximisation step
  - Generate the expected (simulated) map given a set of initial atomic positions, B-factors, and background error.
- Perform expectation step
  - For each atom determine a new desired position and B-factor.
  - Update the background noise term.
- Update the biasing force to encourage atoms towards the new positions.
- Repeat until convergence criteria are satisfied.

### Expectation

The intensity ‘*P*’ due to a given atom ‘*i*’ at a coordinate *v* can be modelled as a Gaussian where $\overrightarrow{{x}_{i}}$, *B*_*i*_ and *Z*_*i*_ are the atoms positions, B-factor and atomic number, respectively:

1 $$P\left(\vec{v},{\vec{x}}_{i},{B}_{i},{Z}_{i}\right)={Z}_{i}{e}^{\frac{{{{{{\rm{|}}}}}}\vec{v}-\vec{{x}_{i}}{{{{{{\rm{|}}}}}}}^{2}}{{{-B}_{i}}^{2}}}$$

For brevity, we abbreviate the above equation for a given atom:

2 $${P}_{i}\left(\vec{v}\right)=P\left(\vec{v},{\vec{x}}_{i},{B}_{i},{Z}_{i}\right)$$

Now, the expected intensity of a given voxel in a cryo-EM map *M*_*s*_ (refered to as the simulated map) is given by the contributions of all *N* atoms with an additional error term *E* which will be introduced later:

3 $${M}_{s}\left(\vec{v}\right)=\mathop{\sum}\limits_{i}^{N}{P}_{i}\left(\vec{v}\right)+E$$

### Maximisation

The maximisation step attempts to determine updated parameters that improve the simulated map in the next ‘expectation’ round. To perform the maximisation step for each atom a responsibility-weighted experimental map ${W}_{i}\left(\vec{v}\right)$ is calculated for each atom. The responsibility for a given atom (${\gamma }_{i}$) is given by:

4 $${\gamma }_{i}\left(\vec{v}\right)=\frac{{P}_{i}\left(\vec{v}\right)}{{M}_{s}\left(\vec{v}\right)}$$

Next, the experimental map ${M}_{e}$ is weighted by this responsibility:

5 $${W}_{i}(\vec{v})={M}_{e}(\vec{v}){\gamma }_{i}(\vec{v})$$

The new position *x*_i_′ of the *i*’th atom is given by the weighted real-space average of the voxels, where $\overrightarrow{v}$ is the real-space position of the voxel.

6 $${x}_{i}^{{\prime} }=\frac{1}{{tot\; mass}}\mathop{\sum}\limits_{{v}\in V}{W}_{i}\left(\vec{v}\right){{{{{\rm{R}}}}}}\left(\vec{v}\right)$$

The new B-factor ${B}_{i}^{{\prime} }$ is given by the weighted variance.

7 $${B}_{i}^{{\prime} }=\frac{1}{{tot\; mass}}\mathop{\sum}\limits_{{v}\in V}{W}_{i}(\vec{v}){{{{{\rm{|}}}}}}\vec{v}-\vec{x}{}_{i}{{{{{{\rm{|}}}}}}}^{2}$$

Due to experimental noise, atomic B-factors are often restrained^10,60^. Here, we apply a simple weighting scheme, where the average B-factor of all atoms in a residue is used to weight the atoms.

The new estimate of the background noise *E*′ is also calculated as the mean of the experimental map weighted by the responsibility of the error, where |*V*| is the total number of voxels. Here, only voxels within 4*σ* of the atoms are included in the calculation. This ensures that the noise term isn’t biased by density values that are not near the refined atoms.

8 $${W}_{{err}}\left(\vec{v}\right)={M}_{e}\left(\vec{v}\right)\frac{E}{{M}_{s}\left(\vec{v}\right)}$$

9 $${E}^{{\prime} }=\frac{1}{{|V|}}\mathop{\sum}\limits_{{v}\in V}{W}_{{err}}(\vec{v})$$

### Defining the fitting potential

After determining improved parameters for the atoms, the force field used to steer them is updated. We consider two methods to improve the fit quality: MD, where the system’s coordinates are integrated over time, taking into account the forces atoms exert on each other; and energy minimisation, where the coordinates of the system are changed to minimise the energy function.

To combine our description of the map with the energy terms that are usually present in force fields, we compute a fictitious force representing the direction of the change in position induced by the Gaussian fitting (for MD). The energy term (*E*_*gmm*_) is defined as:

10 $${E}_{{gmm}}={k}_{{gmm}}\left(1-{e}^{-\frac{{|{\vec{x}}_{i}-{\vec{x}}_{i}^{{\prime} }|}^{2}}{2{B}_{i}^{3}}}\right)$$

where ${k}_{{gmm}}$ is a user-defined constant (we used $1{0}^{5}$ for all refinements in this manuscript), $\vec{{x}_{i}}$ is an atom’s current position, ${\vec{x}}_{i}^{{\prime} }$ is the updated position suggested by the GMM and ${B}_{i}$ is the atomic B-factor.

### Creating composite maps

Given an aligned set of experimental maps with fitted models, we use the mixture modelling formulation we provide to generate a composite map. The responsibilities attributed to each chain of a model can be used to weight their intensities when they are combined into the composite map. Adding the signal from all these maps together typically leads to artefacts at the seams (Fig. 4, Supplementary Fig. 8). To deal with this, the experimental maps are reweighted by the responsibility of the components (rather than the atoms) as per Eq. 4 and then summed together (Supplementary Fig. 12).

The input for the algorithm is a consensus model and multiple pre-aligned composite maps. Given C components each with a corresponding atomic model and an experimental map ${M}_{e,c}$, we create a simulated map ${M}_{c}$ for the component. Here, we use the equation for simulating a map (Eq. 3), but only consider the contributions of the atoms of component C:

11 $${M}_{c}\left(\vec{v}\right)={\Sigma }_{{v}\in V}\left(\vec{v}\right)+{{{{{\rm{E}}}}}}$$

Similarly, the responsibility for a component is determined by normalising it against the simulated map of all components. We retain only the high-resolution regions of these component maps by setting the atomic number to 0 when computing the simulated map for atoms in a given model, provided that the corresponding atom in another component map has a lower B-factor. The responsibility map for a given component, ${\gamma }_{c}$, is computed as follows:

12 $${\gamma }_{c}=\frac{{M}_{c}\left(\vec{v}\right)}{{\sum }_{c}^{C}\,{M}_{c}\left(\vec{v}\right)}$$

Now, the final composite map, ${M}_{C}$, is defined as the sum of all the responsibility-weighted experimental maps.

13 $${M}_{C}\left(\vec{v}\right)=\mathop{\sum}\limits_{c}^{C}{\gamma }_{c}\left(\vec{v}\right){M}_{e,c}\left(\vec{v}\right)$$

### Conformation-based force calculation and MD

OpenMM is used for the conformation-based force calculation and MD^33^. We tested CHARMM36 and AMBER14 in OpenMM (Supplementary Table 3), and they show slight differences in the preferred backbone dihedrals (Supplementary Fig. 13). Although other force fields were available, we used AMBER14 for our runs. We used a GB-Neck2 implicit solvent model^61^ and Langevin integrator with a 0.1 femtosecond timestep to calculate atomic trajectories.

### Running the refinements

Before any positional refinement of a given model, the B-factors for all atoms were refined for 25 iterations. B-factors were capped to a maximum value of 1.5 for membrane proteins and 2.5 for all other models. At each refinement iteration, the simulation was run for 2000-time steps. The CCC was calculated for the updated model, using a global B-factor (set to be equivalent to the global resolution of the cryo-EM map) for map simulation (Eq. 3), and if the CCC did not improve for 5 iterations the refinement was stopped. If this convergence criterium was not met after 300 iterations, the refinement was stopped.

### Local quality of fit (LoQFit)

We implemented a local-fit quality score as part of the TEMPy2 python package. The score – LoQFit – uses an approach similar to a local FSC score for cryo-EM maps^62^ in order to assess the fit quality of a protein model. This local FSC score is calculated for regions defined by a soft-edged spherical mask, centred at the *C*_α_ atom for each residue in the fitted model and applied to both ${M}_{S}$ and ${M}_{E}$. The diameter of this mask is five times the global resolution of the experimental map. We use an FSC threshold of 0.5 to determine the LoQFit score for each residue. To improve the smoothness of the final LoQFit plot, we include an option to estimate the exact frequency at 0.5 correlation between the two maps, using linear interpolation.

We also use SMOCf to estimate the local quality of fit^35^. Briefly, SMOCf uses a local window around each residue, and then computes the Manders overlap coefficient between the simulated observed maps in this region.

### Ensemble algorithm

To compute an ensemble of atomic models that fit the cryo-EM map, we create an ensemble of locally perturbed conformations. This is achieved by sampling the coordinates of each atom from a multivariate Gaussian. The mean value of this Gaussian is set to initial position of each atom, and the covariance matrix is constructed from the shifted B-factors (which are the original B-factors adjusted such that the minimum B-factor is fixed at 0.25). We then locally minimise each model in the ensemble, to keep acceptable stereochemistry.

Following this, we apply an ensemble fitting force and a density-guided force. The ensemble energy term ${E}_{{ens}}$ is defined per atom as:

14 $${E}_{{ens}}=\frac{{k}_{{ens}}}{\sqrt{{2\pi }^{3} \,*\, {{B}_{i}}^{3}}} \,*\, \left( 1 - {e}^{ - \frac{{|} \vec{x}_{i} - \vec{x}_{i}^{{\prime} }{|}^{2}}{{{B}_{i}}^{3}}}\right)$$

where ${E}_{{ens}}$ is a constant (1000 is used for all examples shown in this manuscript), ${B}_{i}$ is the atomic B-factor, $\overrightarrow{x}{\,}_{i}^{{{\hbox{'}}}}$ are the coordinates of the atom after resampling, and ${\overrightarrow{x}}_{i}$ are the coordinates prior to sampling. The energy for the density-guided force is defined as the negative (interpolated) cryo-EM density value at the position of each atom, scaled by a constant ${k}_{{dens}}$, which typically needs to be optimised for each map (values used range between 5 and 200). With these forces applied, we run a short simulation (2000 steps of 0.1 femtoseconds) and minimise using L-BGFS in openMM^33^.

We then generate blurred maps for each conformation in the ensemble, and compute a voxel-based average. To determine the number of models in an ensemble we increase the number of models until there is no increase in CCC. This average blurred map represents the final ensemble average map we use throughout the text.

### RMSF

To compute the RMSF value for our generated ensemble, we first compute the mean structure, and then compute the RMSF using the normal formula. For an ensemble of structures, the residue fluctuation profiles for an ensemble with $N$ models are calculated according to the formula:

15 $${RMSF}=\sqrt{\frac{1}{N}\mathop{\sum}\limits_{j}^{N}\,{\left({x}_{i\left(j\right)}-\left\langle {x}_{i}\right\rangle \right)}^{2}}$$

where ${x}_{i\left(j\right)}$ denotes the position (coordinates) of the i-th Cα atom in the structure of the j-th ensemble model and $\left\langle {x}_{i}\right\rangle$ denotes the averaged position of the i-th Cα atom in all models in the ensemble.

### Local resolution calculations

We used the ResMap method to compute local resolution estimates^63^. ResMap uses local windows of varying size, and statistical tests to determine the most likely resolution for each voxel in the map.

### Generation of benchmark and assessment

Our benchmark is based on the CERES database^38^. We took the corresponding deposited maps and structures from EMDB^64^ and PDB^65^, and the re-refined structures from CERES. Because of the CERES database setup, our benchmark contains maps resolved from 2.1–4.9 Å resolution. We did not include any CERES models that contained stretches of 3 or more consecutive residues with no modelled side chain atoms.

In almost all cases, we assess the goodness-of-fit of models using the CCC with ChimeraX 1.3, using the command *measure correlation*^66^. The exception to this is the results presented in Fig. 3a, and in Fig. S4, in which the CCC was calculated using TEMPy^67^. Simulated maps were generated using TEMPy with a uniform B-factor set to be equivalent to the global resolution value for the cryo-EM map, which was obtained from the EMDB. MolProbity and clash scores were calculated using *phenix.molprobity*^68^, and CaBLAM using *phenix.cablam*^37^.

### Reporting summary

Further information on research design is available in the Nature Portfolio Reporting Summary linked to this article.

### Supplementary information

Supplementary Information

Reporting Summary

## Data Availability

The data that support this study are available from the corresponding authors upon request. We obtained atomic models for refinement from the PDB and CERES, and the corresponding cryo-EM maps from the EMDB. All TEMPy-ReFF refined models described in this paper, alongside the corresponding models from the PDB and CERES, where appropriate, are deposited at the following Zenodo repository: [10.5281/zenodo.8395613]. The AlphaFold2-Multimer predicted model shown in Fig. 7 is also deposited in the same Zenodo repository. The numerical data underlying the plots shown in Figs. 2a, 3a–c, 5d, 6c, 7b are provided as a Source Data file.

## Code availability

TEMPy-ReFF is available at https://www.topf-group.com/tempy-reff.
