# Supplementary material for: Cryo-EM structure and B-factor refinement with ensemble representation
Source: Nat Commun. 2024 Jan 10;15:444. doi: 10.1038/s41467-023-44593-1 (PMC10781738; doi:10.1038/s41467-023-44593-1)
Supplement: Supplementary file 1 — Supplementary Information [file 41467_2023_44593_MOESM1_ESM.pdf]

Supplementary Information for: Cryo-EM structure and B-factor refinement with ensemble representation

Joseph G Beton[1*], Thomas Mulvaney[1*], Tristan Cragnolini[1,2], Maya Topf[1+]

1. Leibniz Institute of Virology (LIV) and Universitätsklinikum Hamburg Eppendorf (UKE), Centre for Structural Systems Biology (CSSB), 22607, Hamburg, Germany
2. Institute of Structural and Molecular Biology, Birkbeck, University of London, London, UK

* Joint authors
\+ corresponding author email: maya.topf@cssb-hamburg.de

EMDB id	CCC	
	Initial	Final
emd_6640	0.428	0.438
emd_10097	0.403	0.430
emd_3488	0.499	0.643
emd_10092	0.514	0.573
emd_30127	0.611	0.636
emd_9102	0.821	0.837
emd_8194	0.627	0.682
emd_3947	0.813	0.829
emd_20278	0.566	0.636
emd_8743	0.655	0.726
emd_20857	0.515	0.607
emd_21430	0.734	0.790
emd_21431	0.681	0.748
emd_0707	0.613	0.698
emd_7875	0.700	0.709
emd_3145	0.552	0.640
emd_3178	0.648	0.717
emd_6272	0.357	0.362

Supplementary Table 1 | Change in CCC upon B-factor refinement. Initial values are listed for a uniform B-factor set to equal the global resolution of the map, final CCC values are listed after 25 B-factor refinement steps.

EMDB id	CCC			Clash score			MolProbity score			CaBLAM outliers (%)		
	Initial	Final	CERES	Initial	Final	CERES	Initial	Final	CERES	initial	Final	CERES
emd_0709	0.762	0.809	0.763	13.89	0.81	15.56	2.12	2.22	2.23	5.8	10.6	5.9
emd_0730	0.782	0.779	0.780	7.65	0.13	7.33	2.38	0.98	1.94	0.6	1.6	0.2
emd_10121	0.587	0.592	0.584	5.62	0.59	10.06	2.41	2.03	2.12	8.0	5.3	7.0
emd_10532	0.679	0.686	0.681	11.35	0.79	21.35	2.30	2.71	2.63	7.6	17.4	8.6
emd_11196	0.575	0.517	0.574	12.31	0.39	9.89	1.87	1.69	1.64	0.8	5.2	0.9
emd_11814	0.735	0.736	0.735	76.67	0.92	70.87	3.06	2.08	2.97	8.2	6.8	6.9
emd_20807	0.658	0.666	0.656	4.10	0.37	8.57	1.67	1.80	1.93	5.3	6.0	4.7
emd_21368	0.512	0.488	0.515	9.91	0.25	9.50	1.87	1.60	1.89	2.5	4.9	2.4
emd_21461	0.644	0.620	0.633	10.73	0.37	8.44	2.13	1.48	2.08	4.5	1.9	4.1
emd_21846	0.645	0.647	0.641	6.31	0.36	7.03	1.46	1.28	1.58	2.0	2.4	1.8
emd_30127	0.695	0.698	0.695	8.99	0.35	9.47	1.77	1.57	1.94	2.0	4.1	1.9
emd_6345	0.798	0.719	0.809	13.77	0.47	15.98	1.65	2.07	1.86	2.7	10.4	1.6
emd_6904	0.686	0.735	0.683	17.87	1.29	28.60	2.45	2.39	2.60	8.8	12.1	7.4
emd_8192	0.847	0.884	0.867	33.50	1.67	26.44	2.47	2.31	2.19	3.6	5.5	3.1
emd_9118	0.536	0.553	0.541	4.24	0.16	8.47	1.82	1.25	1.95	6.3	3.2	2.3
emd_9327	0.609	0.616	0.618	14.31	2.10	19.34	1.78	2.10	1.78	0.6	1.6	0.6

Supplementary Table 2 | Change in scores upon refinement, comparison against CERES

EMDB ID	CCC after refinement with AMBER14	CCC after refinement with CHARMM36
EMD-10097	0.516	0.524
EMD-3488	0.540	0.537
EMD-20278	0.561	0.560
EMD-8743	0.737	0.736
EMD-20857	0.514	0.512

Supplementary Table 3 | Force field comparison. To confirm that no significant bias is introduced by the choice of force field, we tested both AMBER14 and CHARMM36 force fields on our dataset. No significant differences are observed using either AMBER or CHARMM force fields, although the CCCs at the end of refinement were on average slightly higher for AMBER runs.

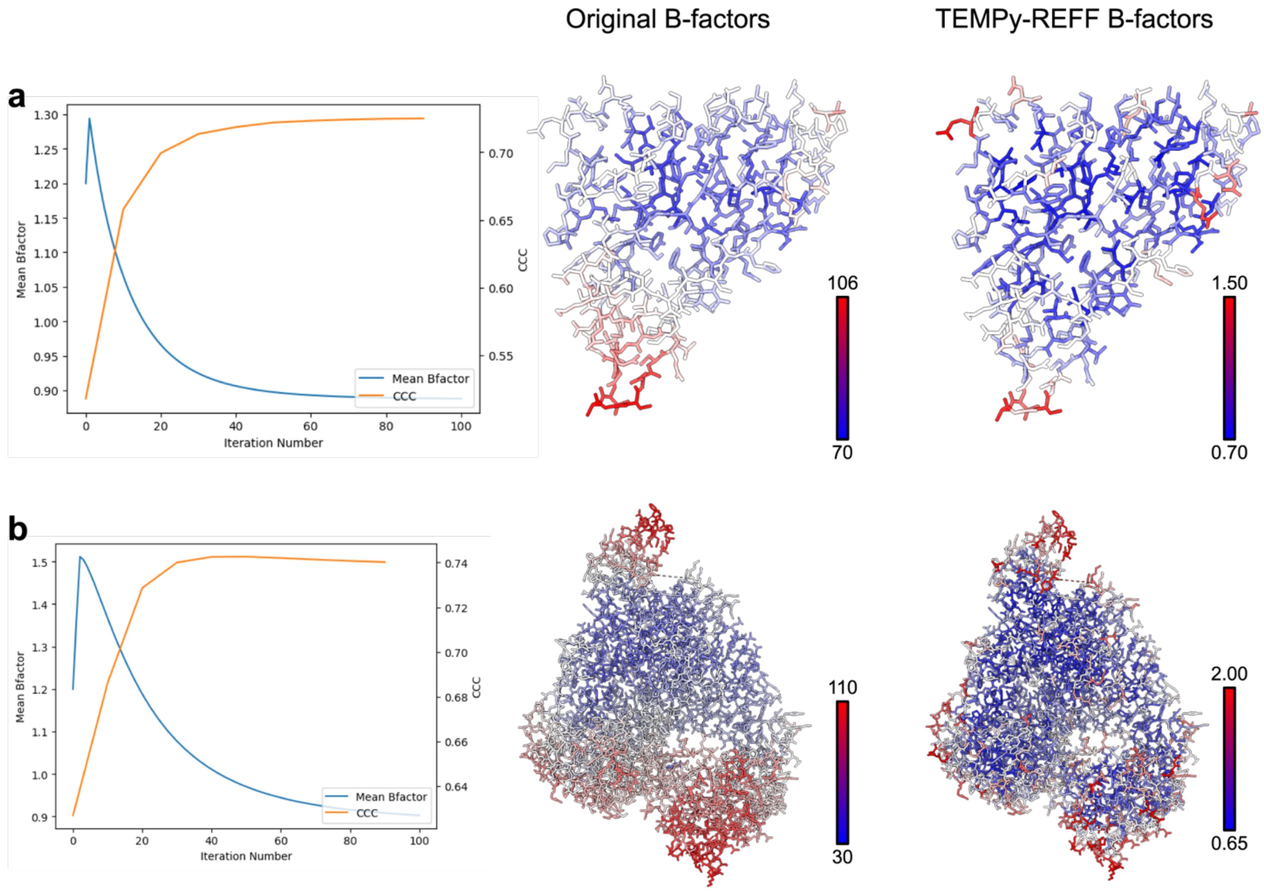

Supplementary Figure 1 | Change in CCC and B-factor during the TEMPy-ReFF refinement procedure a) CCC (orange line) and B-factor (blue line) changes illustrated on EMD-10097; **b)** and EMD-30127. The B-factor converges in around 60 iterations. Significant changes in B-factor distribution can be seen before (middle column) and after refinement (right column).

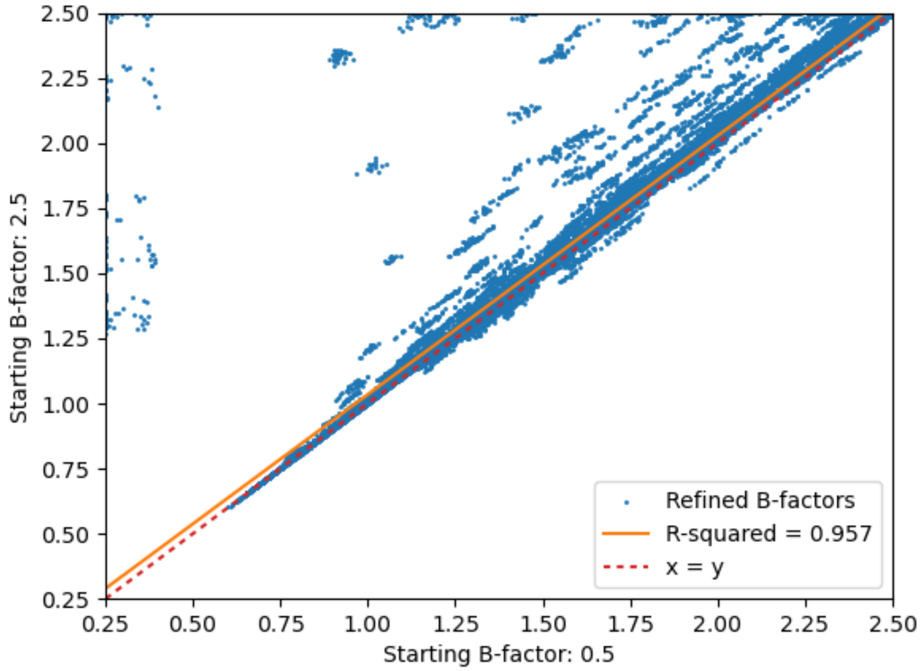

Supplementary Figure 2 | B-factor convergence. The values of the final B-factor (blue points) after TEMPY-REFF refinement, for an initial uniform B-factor of 0.5 vs 2.5. The B-factors are largely independent from the initial value.

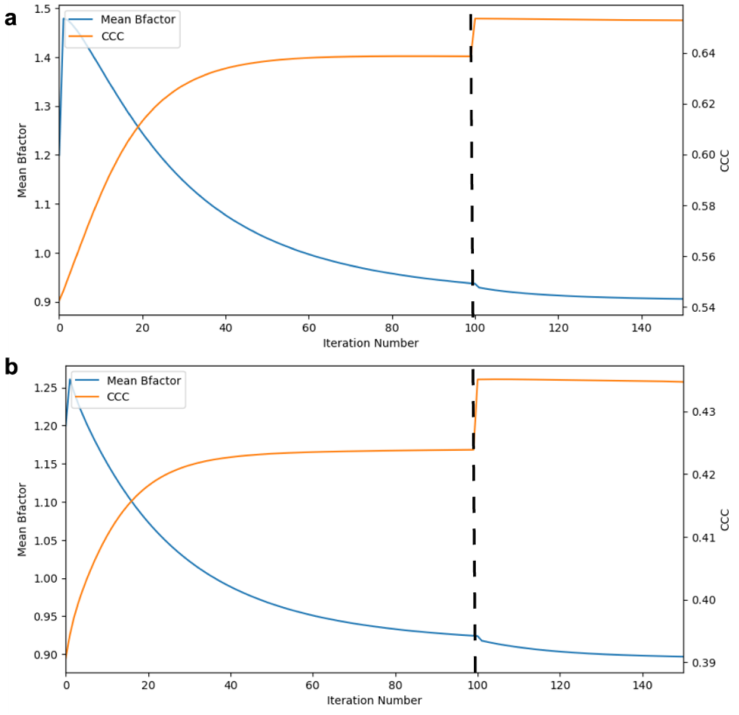

Supplementary Figure 3 | The change in mean B-factor (blue line) and CCC (orange line), before and after position refinement for two assemblies. a, EMD-10097; PDB ID 6S44 **b,** EMD-30127; PDB ID 6M71

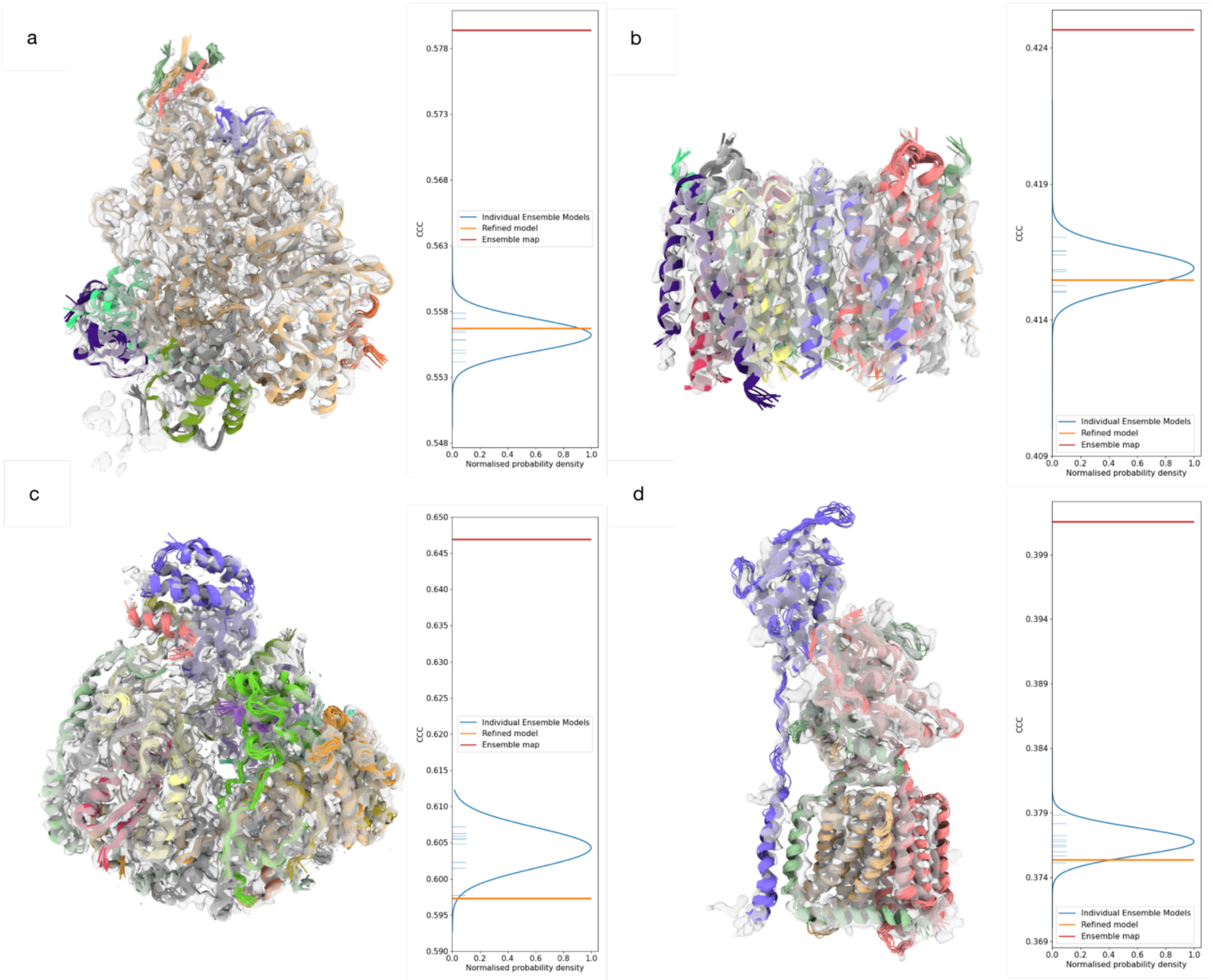

Supplementary Figure 4 | Ensemble generation. a) depiction of an ensemble of structures for SARS-Cov-2 RNA-dependent RNA polymerase at 2.9 Å resolution (EMD-30127, initial model PDB ID: 6M71). The CCC with respect to the map is shown on the right, with the single refinement shown in orange, the each member of the ensemble in blue, and the ensemble average map in red. **b)** As in **a** for otopetrin proton channel Otop3 at 3.22 Å (EMD-9361, initial model PDB ID: 6NF6). **c)** As in a for the apo Machupo virus polymerase at 3.58 Å (EMD-0707, initial model PDB ID: 6KLD). **d)** As in a for human Niemann-Pick C1 at 4.43 Å (EMD-6640, initial model PDB ID: 3JD8).

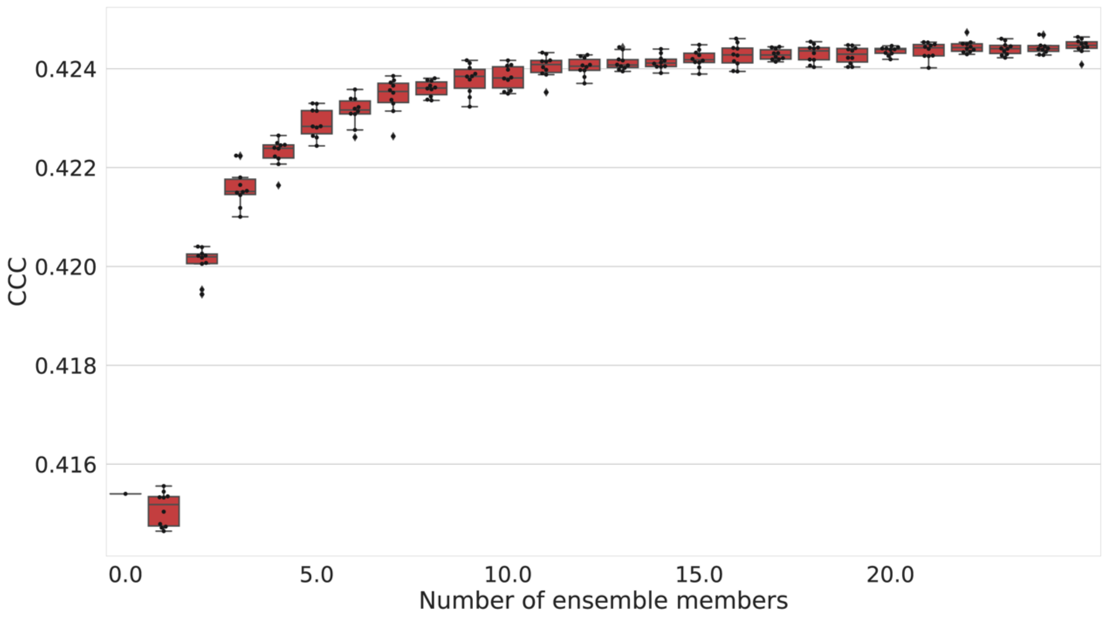

Supplementary Figure 5 | Change in CCC as a function of ensemble size, for the otopetrin proton channel Otop3 at 3.22 Å (EMD-9361, initial model PDB ID: 6NF6). Bootstrap estimates at each size are computed by resampling from the full ensemble. The central line in each box plot defines the median value, the bounds of each box define the upper and lower quartiles and the whiskers define 1.5 times the interquartile range (IQR). Outliers (points outside this 1.5*IQR range) are marked with rhombus symbols.

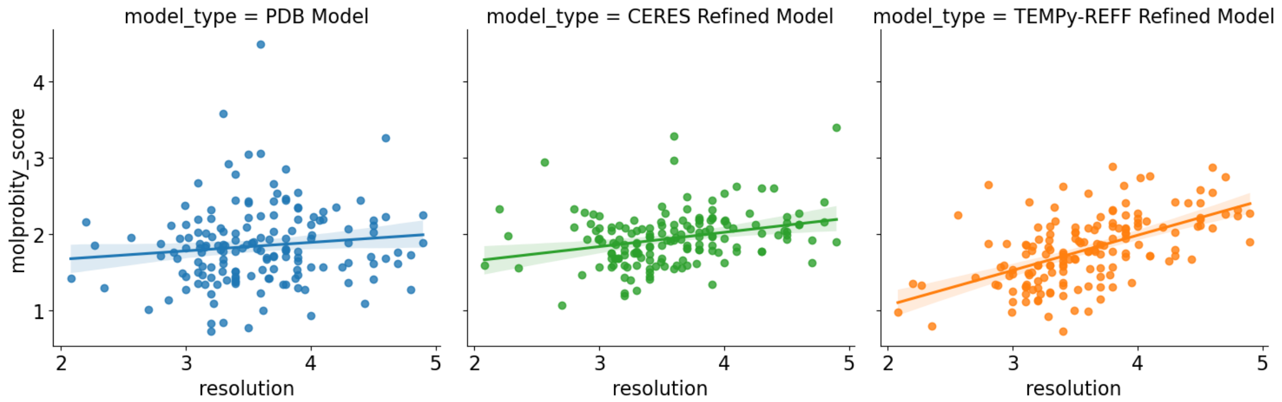

Supplementary Fig 6 | Evaluation of model quality. The model quality (MolProbity score) dependency on resolution for each model refinement method. From left to right: models acquired directly from the PDB (blue points, Pearson's coefficient 0.12), models from the CERES database (green points, Pearson's Coefficient 0.29) and those refined by TEMPy-ReFF (orange points, Pearson's coefficient 0.46).

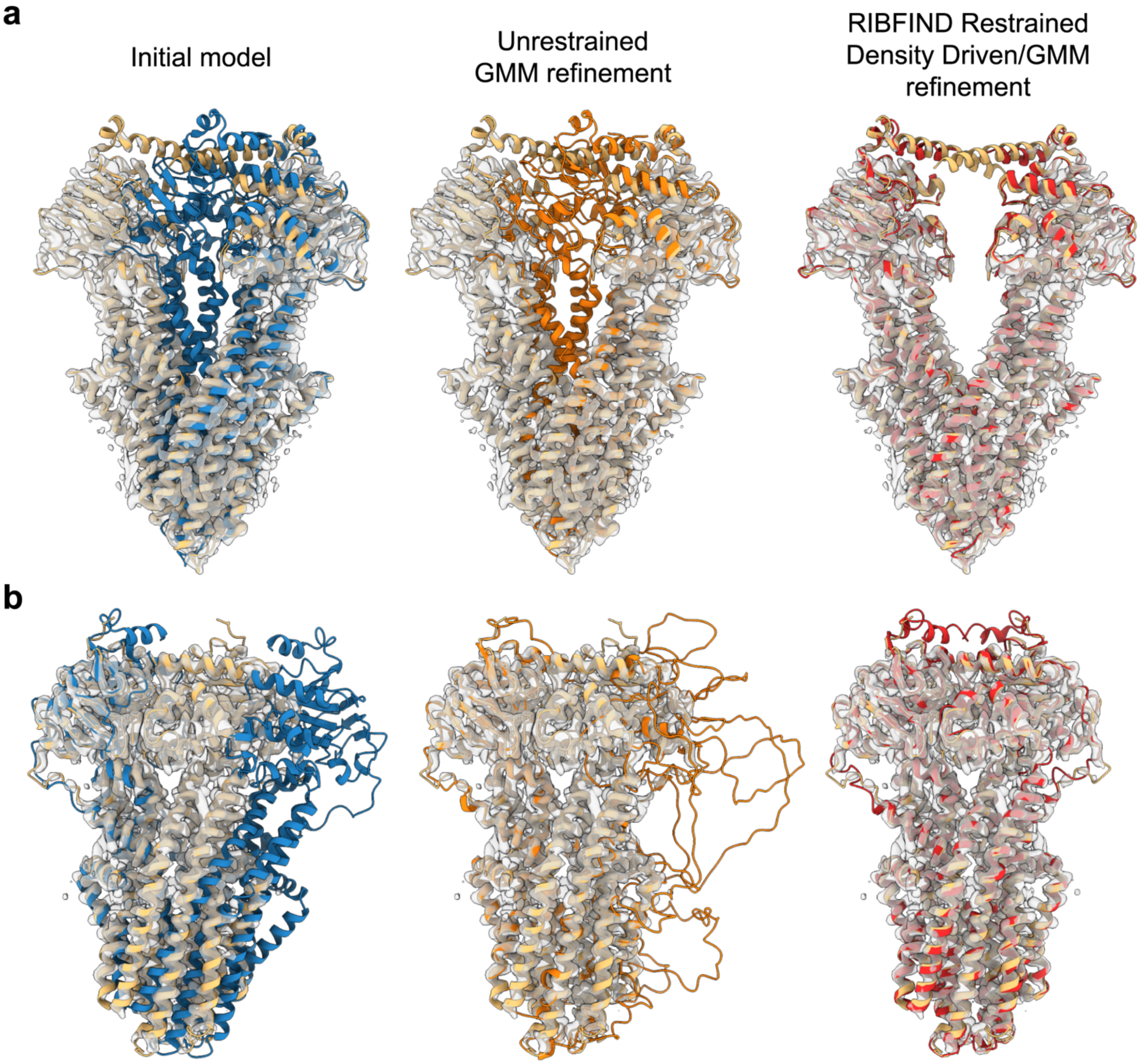

Supplementary Figure 7 | Additional restraints enable large conformational changes. a) TEMPy-ReFF refinement of one state of the Atm1 ABC transporter (PDB ID: 7PSM) into the cryo-EM map of an alternative state (EMDB ID: 13613, 3.3 Å resolution). The cryo-EM map is shown with transparent surface rendering and the corresponding deposited model for EMD-13613 (PDB ID 7PSL, open state) is shown in beige. The starting model for refinement (PDB ID: 7PSM) is shown in blue in the left-hand panel. The TEMPy-ReFF refined model is shown in orange in the central panel. The density-guided RIBFIND2 restrained refinement is shown in red in the right-hand panel. **b)** TEMPy-ReFF refinement of the open conformation of the CGT ABC transporter (PDB ID 7zo8) into the cryo-EM map for the closed conformation (EMDB ID: 14844, 3.5 Å resolution). Colours as per **a**.

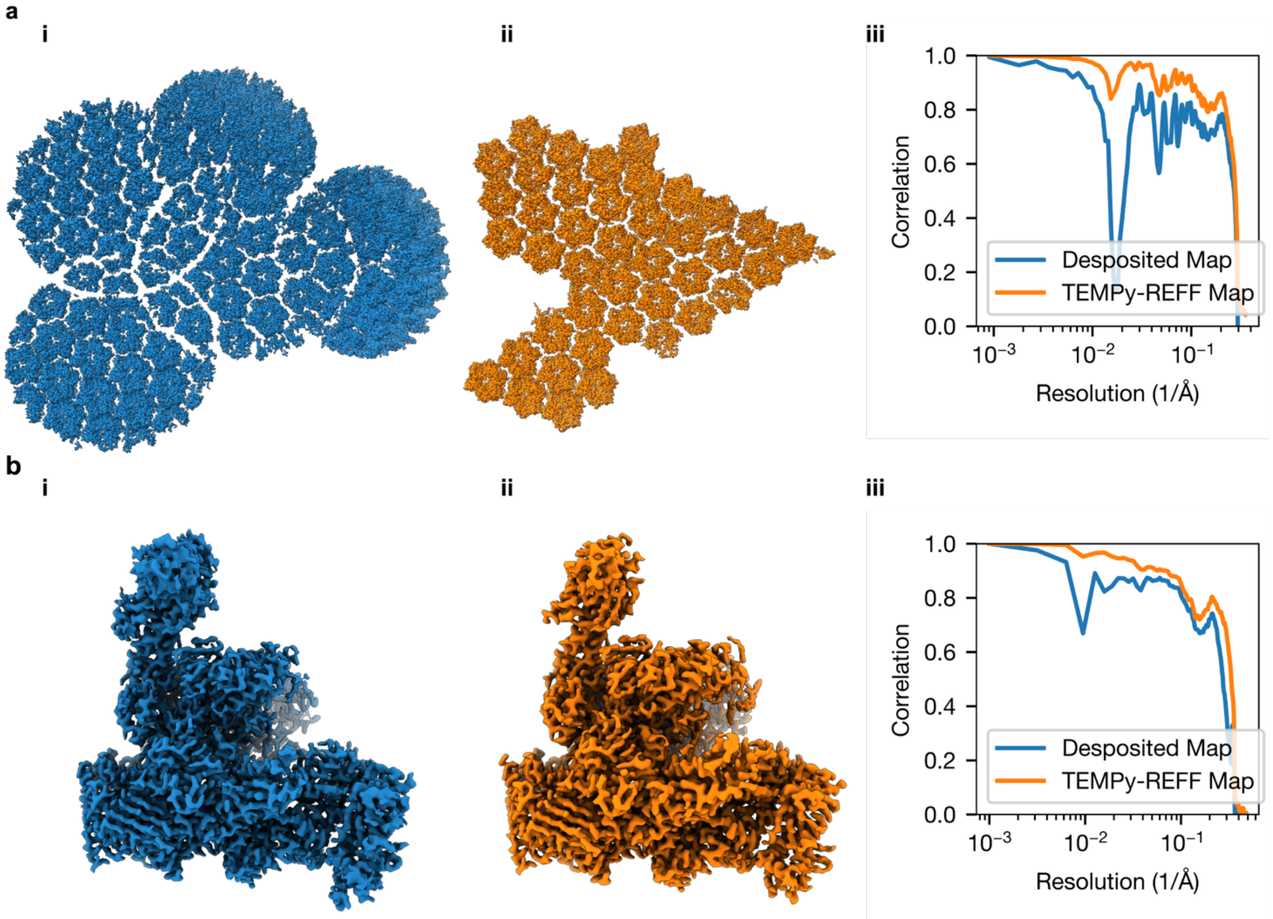

Supplementary Figure 8 | Generating composite maps with TEMPy-ReFF. a) Generating a composite map for the Singapore grouper iridovirus capsid. **i.** Composite map from the EMDB (EMDB ID: 34815), shown with blue surface rendering. **ii.** Composite map generated by TEMPy-ReFF, shown as orange surface rendering. **iii.** The map-model FSC curve for the original composite map (blue line) and the composite map from TEMPy-ReFF (orange line). **b)** Generating a composite map for RNA polymerase II (EMD-12969). The subpanels **(i - iii)** have the same layout and colouring as in **a**.

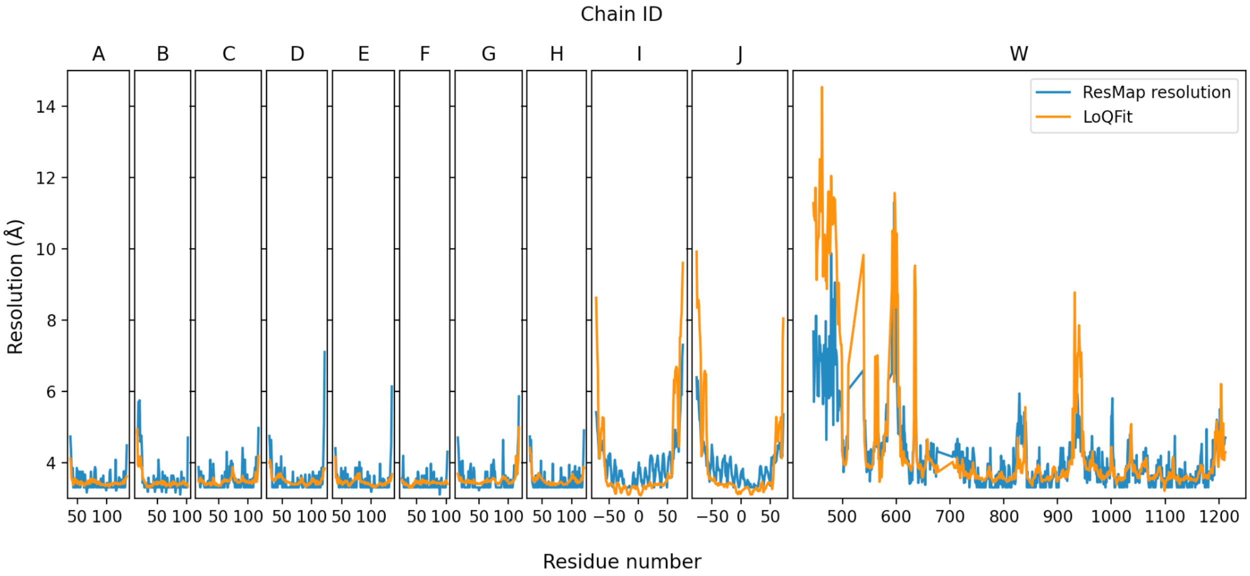

Supplementary Figure 9 | Comparison of LoQFit (orange line) and ResMap local resolution (blue line) for the nucleosome-CHD4 complex. Both follow very similar trends, with regions of locally higher and lower resolutions in agreement between the methods. (To obtain a local resolution along the chain for ResMap, we compute the average local resolution of the voxels in which the given residue is contained).

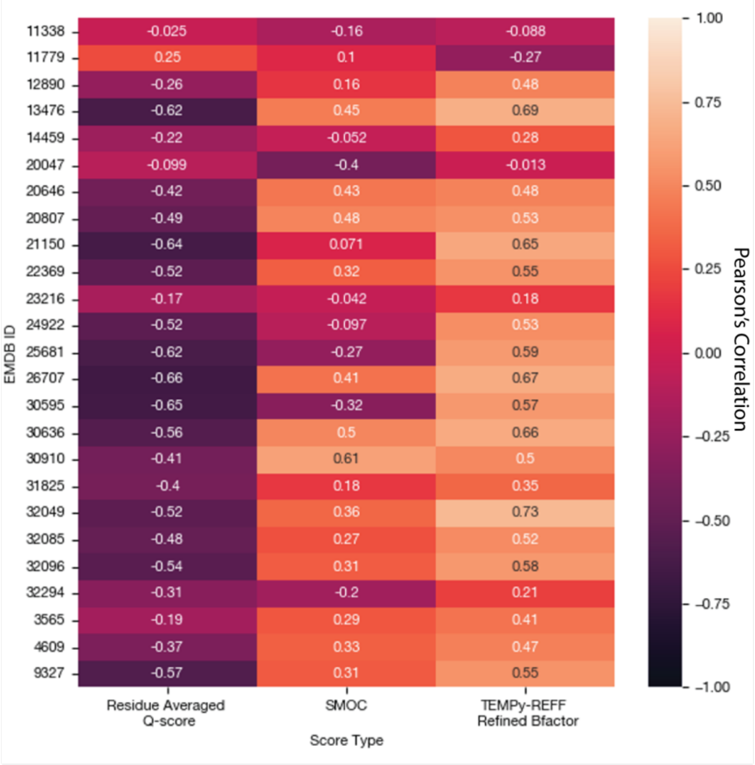

Supplementary Figure 10 | Correlation between LoQFit and other local scoring functions. The correlation between residue scores calculated with LoQFit and Q-score$_{avg}$ (calculated using the ChimeraX plugin), SMOCf and the average TEMPy-ReFF refined B-factor for each residue. The Pearson's Coefficient was calculated between LoQFit and each respective score across 50 TEMPy-ReFF refined models randomly chosen from the overall CERES benchmark, but here only the correlation between LoQFit and the respective scores for 25 randomly selected single models are shown.

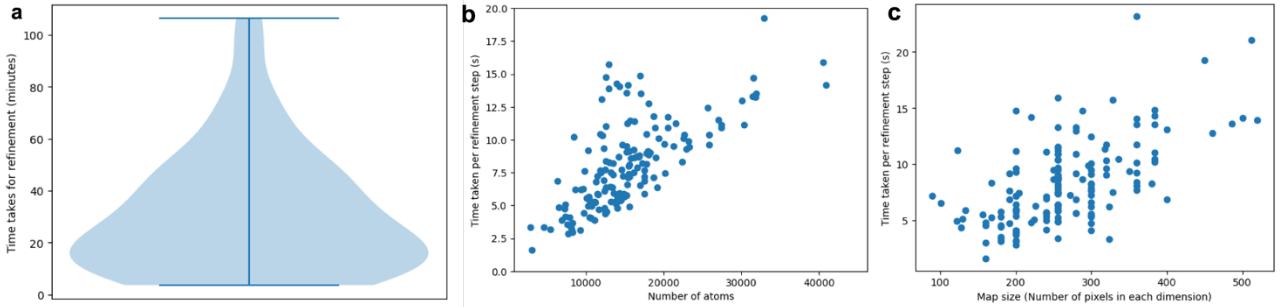

Supplementary Fig 11: Relationship between map and model size and speed of refinement. **a)** Violin plot showing the overall time taken for refinement of all models used in the CERES benchmark. Scatter plots showing the average time taken for each step of the refinement for each model in our benchmark relative to **b)** the number of atoms in the model (Pearson's coefficient 0.67) and **c)** the size of the cryo-EM map (Pearson's coefficient 0.60).

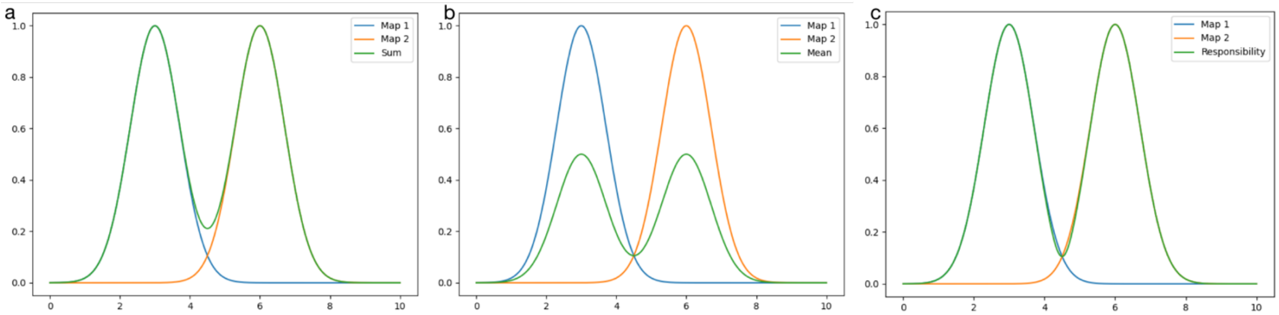

Supplementary Figure 12 | Schematics of map composition with responsibilities. We use two Gaussians to illustrate the map composition process (blue and orange). Each Gaussian is an idealised representation of a focused map. Three protocols for combining those maps into a single composed map are shown, with the resulting map in green. **a)** Summing the intensities from multiple maps will overcount the transition regions (creating a ‘seam’). **b)** Averaging the maps will undercount the peak intensities **c)** Using responsibilities, the weighting of each map switches smoothly from map 1, to equal weighting at the seam, to map 2. This results in properly accounting for the intensities across the whole domain.

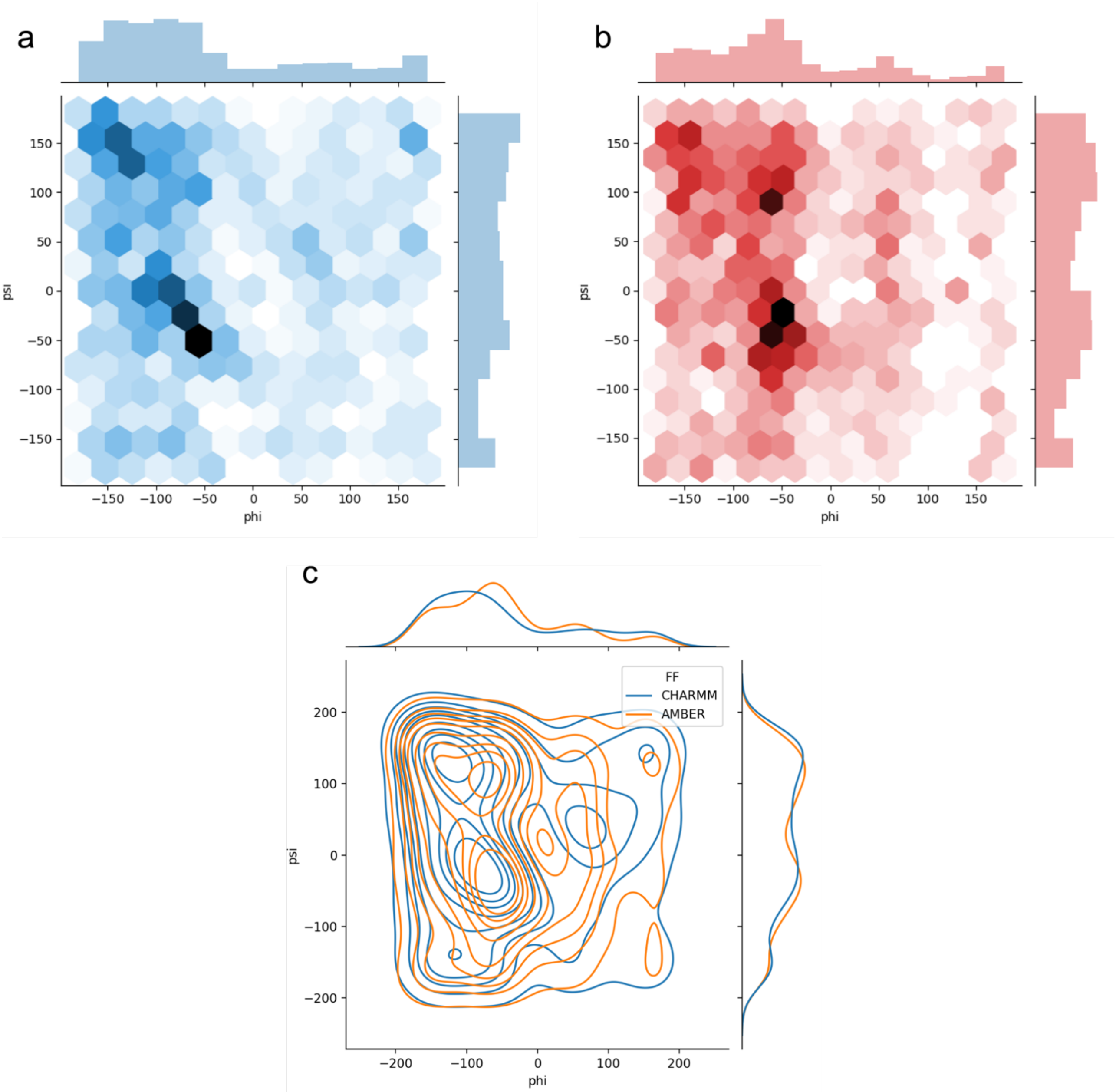

Supplementary Figure 13 | Ramachandran plot for **a)** CHARMM36 and **b)** AMBER14: although similar, both forcefields exhibit slightly different preferences regarding dihedral combinations; the centres are slightly offset (most visible in **c**), and differently populated.
