# Supplementary material for: Cryo-EM structure and B-factor refinement with ensemble representation
Source: Nat Commun. 2024 Jan 10;15:444. doi: 10.1038/s41467-023-44593-1 (PMC10781738; doi:10.1038/s41467-023-44593-1)
Supplement: Supplementary file 2 — Reporting Summary [file 41467_2023_44593_MOESM2_ESM.pdf]

nature portfolio

Corresponding author(s): Maya Topf

Last updated by author(s): Dec 11, 2023

Reporting Summary

Nature Portfolio wishes to improve the reproducibility of the work that we publish. This form provides structure for consistency and transparency in reporting. For further information on Nature Portfolio policies, see our Editorial Policies and the Editorial Policy Checklist.

Statistics

For all statistical analyses, confirm that the following items are present in the figure legend, table legend, main text, or Methods section.

n/a	Confirmed	
☐	☒	The exact sample size (*n*) for each experimental group/condition, given as a discrete number and unit of measurement
☒	☐	A statement on whether measurements were taken from distinct samples or whether the same sample was measured repeatedly
☒	☐	The statistical test(s) used AND whether they are one- or two-sided<br>*Only common tests should be described solely by name; describe more complex techniques in the Methods section.*
☒	☐	A description of all covariates tested
☒	☐	A description of any assumptions or corrections, such as tests of normality and adjustment for multiple comparisons
☐	☒	A full description of the statistical parameters including central tendency (e.g. means) or other basic estimates (e.g. regression coefficient) AND variation (e.g. standard deviation) or associated estimates of uncertainty (e.g. confidence intervals)
☒	☐	For null hypothesis testing, the test statistic (e.g. *F*, *t*, *r*) with confidence intervals, effect sizes, degrees of freedom and *P* value noted<br>*Give P values as exact values whenever suitable.*
☒	☐	For Bayesian analysis, information on the choice of priors and Markov chain Monte Carlo settings
☒	☐	For hierarchical and complex designs, identification of the appropriate level for tests and full reporting of outcomes
☐	☒	Estimates of effect sizes (e.g. Cohen's *d*, Pearson's *r*), indicating how they were calculated

Our web collection on statistics for biologists contains articles on many of the points above.

Software and code

Policy information about availability of computer code

Data collection	We used a custom script to automate data collection from the PDB, EMDB and CERES databases. This script is included in the Zenodo repository: 10.5281/zenodo.8395614. We used AlphaFold-Multimer version 2.3.2 to generate a predicted model.
Data analysis	We used our TEMPy-ReFF software which is available at https://gitlab.com/topf-lab/tempy-reff. We assessed the quality of these models using TEMPy (version 2.1), ChimeraX (version 1.3) and Phenix (version 1.20.1-4487) We also used Q-score ChimeraX plug-in (version 1.0), RIBFIND (version 2.0) and Resmap (version 1.1.4).

For manuscripts utilizing custom algorithms or software that are central to the research but not yet described in published literature, software must be made available to editors and reviewers. We strongly encourage code deposition in a community repository (e.g. GitHub). See the Nature Portfolio guidelines for submitting code & software for further information.

Data

Policy information about availability of data

All manuscripts must include a data availability statement. This statement should provide the following information, where applicable:

- Accession codes, unique identifiers, or web links for publicly available datasets
- A description of any restrictions on data availability
- For clinical datasets or third party data, please ensure that the statement adheres to our policy

We obtained atomic models for refinement from the PDB and CERES, and the corresponding cryo-EM maps from the EMDB. The TEMPy-ReFF refined models

described in this paper, alongside the corresponding models from the PDB and CERES, are deposited with the following DOI: https://doi.org/10.5281/zenodo.8395613. The numerical data underlying the plots shown in figures 2a, 3a-c, 5d, 6c, 7b are provided as a Source Data file.

Research involving human participants, their data, or biological material

Policy information about studies with human participants or human data. See also policy information about sex, gender (identity/presentation), and sexual orientation and race, ethnicity and racism.

Reporting on sex and gender	Not applicable
Reporting on race, ethnicity, or other socially relevant groupings	Not applicable
Population characteristics	Not applicable
Recruitment	Not applicable
Ethics oversight	Not applicable

Note that full information on the approval of the study protocol must also be provided in the manuscript.

Field-specific reporting

Please select the one below that is the best fit for your research. If you are not sure, read the appropriate sections before making your selection.

☒ Life sciences ☐ Behavioural & social sciences ☐ Ecological, evolutionary & environmental sciences

For a reference copy of the document with all sections, see nature.com/documents/nr-reporting-summary-flat.pdf

Life sciences study design

All studies must disclose on these points even when the disclosure is negative.

Sample size	229 atomic models from CERES and the PDB. This was the maximum dataset size that was feasible with our computing resources.
Data exclusions	Atomic models from CERES and/or PDB with missing side-chains were excluded as it would have been unfair to compare the our complete models against these.
Replication	Our software is openly available to allow the community to reproduce results.
Randomization	The models used in our benchmark were randomly selected from all models present in the CERES database.
Blinding	Blinding is not relevant to our study as the refinement workflow is totally automated

Reporting for specific materials, systems and methods

We require information from authors about some types of materials, experimental systems and methods used in many studies. Here, indicate whether each material, system or method listed is relevant to your study. If you are not sure if a list item applies to your research, read the appropriate section before selecting a response.

Materials & experimental systems

n/a	Involved in the study	
☒	☐	Antibodies
☒	☐	Eukaryotic cell lines
☒	☐	Palaeontology and archaeology
☒	☐	Animals and other organisms
☒	☐	Clinical data
☒	☐	Dual use research of concern
☒	☐	Plants

Methods

n/a	Involved in the study	
☒	☐	ChIP-seq
☒	☐	Flow cytometry
☒	☐	MRI-based neuroimaging
